# Gene Expression (mRNA) Markers for Differentiating between Malignant and Benign Follicular Thyroid Tumours

**DOI:** 10.3390/ijms18061184

**Published:** 2017-06-02

**Authors:** Bartosz Wojtas, Aleksandra Pfeifer, Malgorzata Oczko-Wojciechowska, Jolanta Krajewska, Agnieszka Czarniecka, Aleksandra Kukulska, Markus Eszlinger, Thomas Musholt, Tomasz Stokowy, Michal Swierniak, Ewa Stobiecka, Ewa Chmielik, Dagmara Rusinek, Tomasz Tyszkiewicz, Monika Halczok, Steffen Hauptmann, Dariusz Lange, Michal Jarzab, Ralf Paschke, Barbara Jarzab

**Affiliations:** 1 Department of Nuclear Medicine and Endocrine Oncology, Maria Sklodowska-Curie Institute—Oncology Center, Gliwice Branch, Wybrzeze Armii Krajowej 15, 44-101 Gliwice, Poland; Bartosz.Wojtas@io.gliwice.pl (B.W.); Aleksandra.Pfeifer@io.gliwice.pl (A.P.); Malgorzata.Oczko-Wojciechowska@io.gliwice.pl (M.O.-W.); Jolanta.Krajewska@io.gliwice.pl (J.K.); Aleksandra.Kukulska@io.gliwice.pl (A.K.); tomasz.stokowy@k2.uib.no (T.S.); michal.swierniak@wum.edu.pl (M.S.); Dagmara.Rusinek@io.gliwice.pl (D.R.); Tomasz.Tyszkiewicz@io.gliwice.pl (T.T.); Monika.Kowal@io.gliwice.pl (M.H.); 2 Laboratory of Molecular Neurobiology, Neurobiology Center, Nencki Institute of Experimental Biology, Pasteura 3, 02-093 Warsaw, Poland; 3 Faculty of Automatic Control, Electronics and Computer Science, Silesian University of Technology, Akademicka 2A, 44-100 Gliwice, Poland; 4 The Oncologic and Reconstructive Surgery Clinic, Maria Sklodowska-Curie Institute—Oncology Center, Gliwice Branch, Wybrzeze Armii Krajowej 15, 44-101 Gliwice, Poland; Agnieszka.Czarniecka@io.gliwice.pl; 5 Department of Oncology & Arnie Charbonneau Cancer Institute, Cumming School of Medicine, University of Calgary, Calgary, AB T2N 4N1, Canada; markus.eszlinger1@ucalgary.ca; 6 Department of General, Visceral, and Transplantation Surgery, University Medical Center of the Johannes Gutenberg University, D55099 Mainz, Germany; musholt@uni-mainz.de; 7 Department of Clinical Science, University of Bergen, 5020 Bergen, Norway; 8 Genomic Medicine, Department of General, Transplant, and Liver Surgery, Medical University of Warsaw, Zwirki i Wigury 61, 02-093 Warsaw, Poland; 9 Tumor Pathology Department, Maria Sklodowska-Curie Institute—Oncology Center, Gliwice Branch, Wybrzeze Armii Krajowej 15, 44-101 Gliwice, Poland; Ewa.Stobiecka@io.gliwice.pl (E.S.); Ewa.Chmielik@io.gliwice.pl (E.C.); dlange693@gmail.com (D.L.); 10 Department of Pathology, Martin Luther University Halle-Wittenberg, 06108 Halle (Saale), Germany; steffen.hauptmann@patho-ao.de; 11 III Department of Radiotherapy and Chemotherapy, Maria Sklodowska-Curie Institute—Oncology Center, Gliwice Branch, Wybrzeze Armii Krajowej 15, 44-101 Gliwice, Poland; Michal.Jarzab@io.gliwice.pl; 12 Division of Endocrinology, Departments of Medicine, Pathology, Biochemistry & Molecular Biology, and Oncology, and Arnie Charbonneau Cancer Institute, Cumming School of Medicine, University of Calgary, Calgary, Alberta T2N 4N1, Canada; ralf.paschke@ucalgary.ca

**Keywords:** follicular thyroid adenoma, follicular thyroid cancer, gene expression, microarray, meta-analysis

## Abstract

Distinguishing between follicular thyroid cancer (FTC) and follicular thyroid adenoma (FTA) constitutes a long-standing diagnostic problem resulting in equivocal histopathological diagnoses. There is therefore a need for additional molecular markers. To identify molecular differences between FTC and FTA, we analyzed the gene expression microarray data of 52 follicular neoplasms. We also performed a meta-analysis involving 14 studies employing high throughput methods (365 follicular neoplasms analyzed). Based on these two analyses, we selected 18 genes differentially expressed between FTA and FTC. We validated them by quantitative real-time polymerase chain reaction (qRT-PCR) in an independent set of 71 follicular neoplasms from formaldehyde-fixed paraffin embedded (FFPE) tissue material. We confirmed differential expression for 7 genes (*CPQ*, *PLVAP*, *TFF3*, *ACVRL1*, *ZFYVE21*, *FAM189A2*, and *CLEC3B*). Finally, we created a classifier that distinguished between FTC and FTA with an accuracy of 78%, sensitivity of 76%, and specificity of 80%, based on the expression of 4 genes (*CPQ*, *PLVAP*, *TFF3*, *ACVRL1*). In our study, we have demonstrated that meta-analysis is a valuable method for selecting possible molecular markers. Based on our results, we conclude that there might exist a plausible limit of gene classifier accuracy of approximately 80%, when follicular tumors are discriminated based on formalin-fixed postoperative material.

## 1. Introduction

Follicular neoplasms are the most controversial area in the thyroid pathology. According to World Health Organization (WHO) follicular adenoma is a benign, encapsulated tumor of the thyroid showing follicular cell differentiation [1]. This tumor demonstrates no evidence of capsular or vascular invasion. Follicular carcinoma is a malignant tumor showing evidence of follicular cell differentiation. The distinction between follicular adenoma and carcinoma is based on the presence of capsular and/or vascular invasion. Capsular invasion is defined by tumor penetration through the entire thickness of the capsule [1]. The invading tumor nests should present a connection with main tumor mass. The interpretation of capsular invasion may be sometimes problematic. According to the literature data and our experience there is a group of patients with only partial capsular invasion but presenting metastases of follicular carcinoma [2]. Yamashina analyzed entire circumference of tumor capsules of follicular neoplasms and observed that tumors with only capsular invasion in initial sections also presented vascular invasion on additional slices adjacent to tumor capsule [3]. Therefore it would be advisable to evaluate gene expression of follicular adenomas and follicular carcinomas.

Between 2000 and 2014, numerous studies have investigated the gene expression (mRNA) profile that would differentiate follicular thyroid adenoma (FTA) from follicular thyroid cancer (FTC) to improve the diagnostic process and to find features of follicular thyroid tumours important for malignant potential (Table S1) [4,5,6,7,8,9,10,11,12,13,14,15,16,17]. However, reproducibility of results obtained between mentioned publications was rather low. This could be a consequence of slight molecular differences between FTC and FTA [18,19] or the insufficient sample size used in these studies. Genetic alterations, such as *RAS* gene family somatic mutations or *PAX8/PPARG* translocations, although very promising in initial studies, were not found to be specific for follicular carcinoma, as these genetic alterations occurred in both FTCs and FTAs with similar frequencies [20,21,22]. These doubts stimulated us to carry on a meta-analysis.

In our study, we also raised the problem of oncocytic tumors. WHO involves oncocytic thyroid carcinoma (OTC) to FTC and respectively oncocytic adenoma to FTA. Oncocytic tumors (Hurthle cell tumors) are believed to have a different gene expression profile [23,24]. Ganly et al. demonstrated on the basis of mutational, transcriptional, and copy number profiles that Hurthle cell carcinoma was a unique thyroid cancer distinct from papillary thyroid cancer (PTC) and FTC [24].

In the present study we decided to base on FTC definition, proposed by the WHO. Nevertheless, we tried to check whether an inclusion of oncocytic follicular carcinoma does not influence on molecular markers selection. OTC is composed predominantly of oncocytic cells. These tumors are associated with a higher frequency of extrathyroidal extension, local recurrence, nodal metastases in more than 30% of cases and occasionally distant lung and bone metastases [1]. Compared with conventional follicular carcinomas, oncocytic follicular carcinomas are more aggressive [1]. Therefore, it may be reasonable to involve oncocytic feature in our analysis.

Most recent thyroid studies have focused on identifying molecular markers supporting pre-operative FNAB examination to exclude malignancy [25,26]. In 2010, Chudova et al. published a study focused on determining the general preoperative distinction between benign and malignant thyroid nodules, which appeared promising and resulted in the establishment of the Afirma classifier [25]. Our approach, used in the present study, is different.

**Figure 1:**
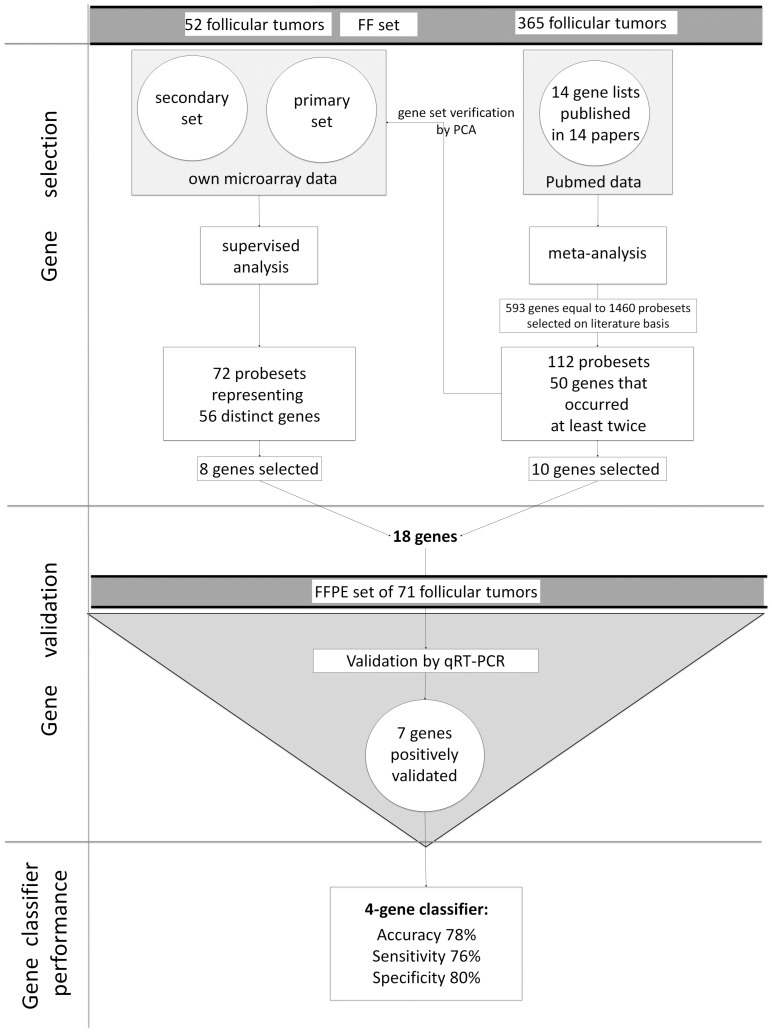
Presentation of a study scheme.

In our study, we utilised two different approaches to select new gene-expression markers for differentiating between FTC and FTA tumours. We performed a two-step analysis: first a statistical testing of a large gene expression microarray dataset of FTC and FTA previously generated in our laboratory [18,27], and next, a meta-analysis of all available datasets, to select the most robustly represented markers [4,5,6,7,8,9,10,11,12,13,14,15,16,17] (Figure 1). Such approach allowed us to select independent genes coming from own dataset and from a meta-analysis. Meta-analysis by combining the results of various studies enabled us to draw common conclusions. The results of both analyses were further validated by quantitative real-time polymerase chain reaction (qRT-PCR) using an independent dataset of follicular tumours.

## 2. Results

### 2.1. Supervised Analysis of Gene Expression Microarrays

Fresh-frozen (FF) material from 52 tumors (27 FTC, 25 FTA) was used for our gene expression microarray experiment and divided into primary and secondary sets. The primary one was considered as highly reliable dataset and contained all samples that were independently and concordantly diagnosed by two thyroid pathology experts. The secondary set contained samples that were diagnosed by only one expert, equivocal samples diagnosed by two experts and a one sample that was discordantly diagnosed according to malignancy.

To select potential molecular markers useful in the distinction between FTA and FTC, we considered genes that were differentially expressed in the primary and secondary microarray datasets. We compared the lists of genes obtained in the analysis of the primary and the secondary sets and selected only those that were significant in both sets. Our secondary microarray set contained borderline and ambiguous cases, and we established genes as valuable and characteristic when they were also differentially expressed in this set.

There were 72 differentially expressed probe sets (representing 56 distinct genes) and 6 non-annotated probe sets. Eight genes were selected (*ACVRL1*, *CLEC3B*, *DIP2B*, *GABARAPL2*, *ZFYVE21*, *LIMK2*, *ZMYND11*, and *MAFB*) for validation by qRT-PCR (Table 1). Those genes were characterised by low false discovery rate (FDR) value, high fold-change, and from our point of view, they could be biologically interesting. Another selection criterion was that these genes were not previously validated as markers differentiating FTCs from FTAs.

As it has been shown that the oncocytic FTC is a unique thyroid cancer distinct from non-oncocytic FTC [24] we decided to perform an additional analysis. We excluded oncocytic samples from microarray dataset (just for the sake of this particular analysis) and evaluated the significance of eight selected genes in the dataset comprising of non-oncocytic samples only to investigate the differences between FTC and FTA (7 FTC and 11 FTA). All these genes showed significant differential expression between FTC and FTA in this dataset (Table 1).

### 2.2. Meta-Analysis

We included 14 papers in which the difference in gene expression between FTC and FTA was assessed by a high throughput method (expression microarrays, serial analysis of gene expression (SAGE), high-throughput differential screening by serial analysis of gene expression (HDSS), adapter-tagged competitive polymerase chain reaction (ATAC-PCR)) (Table S1). The papers were published during the years 2000–2014 and in total 365 samples (201 FTA and 164 FTC) were analyzed.

All reported genes differentiating FTC and FTA were extracted from these publications. We identified 600 genes reported in at least one publication, while 57 genes were reported in more than one publication. Fifty out of those 57 genes were reported with concordant direction of change (Table 2). Seven genes (*CA4*, *EGR2*, *FAM189A2*, *KCNAB1*, *CPQ*, *SLC26A4*, *TFF3*) were reported in 3 publications. Two of these genes (*CA4*, and *KCNAB1*) were already evaluated by qRT-PCR as described in our previous study [27]. 

Among the genes selected based on the meta-analysis, ten genes were chosen for qRT-PCR validation. We chose five down-regulated genes that occurred in three papers (*EGR2*, *FAM189A2*, *SLC26A4*, *TFF3*, *CPQ*), four up-regulated genes that occurred in two papers (*CKS2*, *GDF15*, *ASNS*, *DDIT3*), and one down-regulated gene that occurred in two papers and simultaneously showed significant differences in expression in our primary microarray dataset (*PLVAP*).

### 2.3. Principal Component Analysis

**Figure 2:**
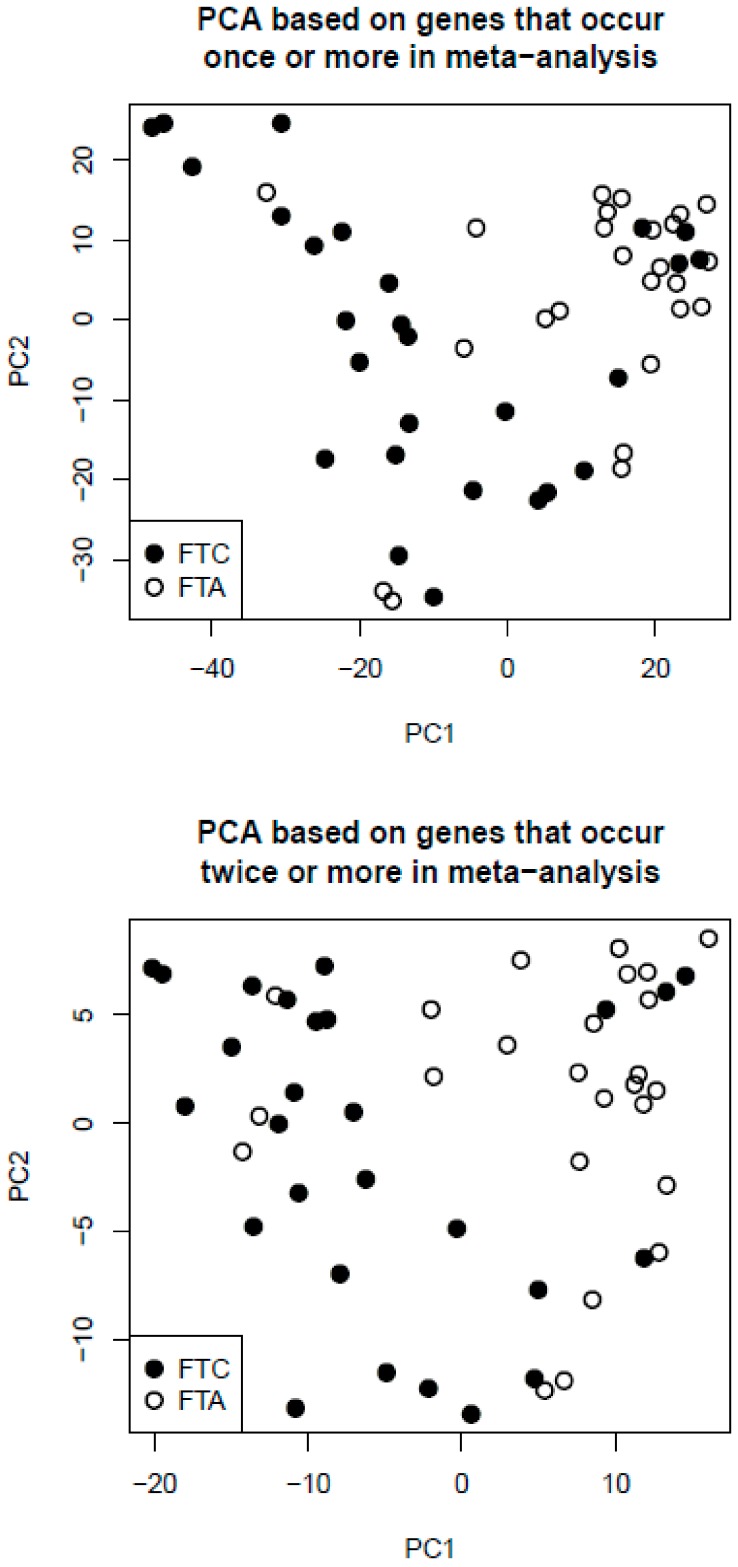
Principal component analysis (PCA) results. PCA plots of samples from our own microarray dataset, based on genes selected in the meta-analysis that occurred in at least one paper (**upper** plot) or at least two papers (**lower** plot).

We selected 593 genes that occurred at least once in the meta-analysis (excluding seven genes with discordant direction of change). We identified HG-U133 PLUS 2 Affymetrix microarray probe sets for these genes. There were 1460 such probe sets (for some genes there was more than one probe set). Next, we performed PCA of our own microarray samples (combined primary and secondary dataset) based on these 1460 probe sets (Figure 2, upper plot). Similarly, we selected 50 genes that occurred at least twice in investigated papers (excluding the genes with discordant direction of change). We identified HG-U133 PLUS 2 Affymetrix microarray probe sets for these genes. There were 112 such probe sets. We performed PCA based on these 112 probe sets (Figure 2, lower plot). Although gene selection was independent of the microarray dataset, we achieved good discrimination of benign and malignant tumors in both analyses. However, the discrimination was not perfect, because a few FTA samples clustered with the FTC group, and a few FTC samples clustered with the FTA group.

### 2.4. qRT-PCR Validation

qRT-PCR was used to validate 18 genes selected based on the analysis of our own microarray dataset and the meta-analysis (Table 3). *GABARAPL2*, *DDIT3*, and *SLC26A4* amplification was not possible in the FFPE samples (probably due to low endogenous expression), and therefore, it was excluded from validation.

**Figure 3:**
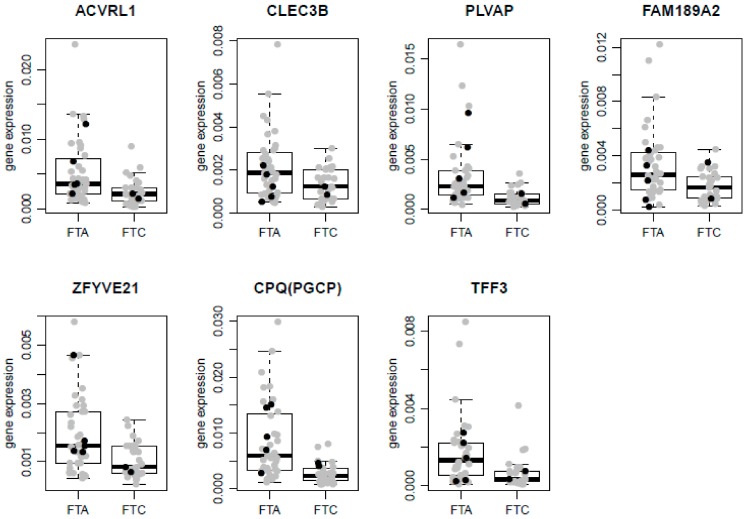
The normalized relative expression levels of positively validated genes in the FFPE dataset of 69 samples. Boxplots superimposed with scatterplots are shown. The line inside each box corresponds to median. Upper and lower edges of boxes correspond to first (Q1) and third (Q3) quartiles, respectively. The whiskers extend to smallest and largest observations within 1.5 times interquartile range (IQR) from the box. Black dots represent *RAS* mutation carrying samples, and grey dots represent samples without *RAS* mutation.

Log-transformed expression levels of the remaining 15 genes were analysed using the Student’s *t*-test (Table 3). Two FTC samples were extreme outliers (the expression was higher than third quartile (Q3) + 6 × interquartile range (IQR)) in two distinct genes. These samples were excluded from further analysis. Differential expression of *CPQ*, *PLVAP*, *TFF3*, *ACVRL1*, *ZFYVE21*, *FAM189A2*, and *CLEC3B* was confirmed by qRT-PCR contrary to the expression of *ZMYND11*, *LIMK2*, *DIP2B*, *MAFB*, *CKS2*, *ASNS*, *EGR2*, and *GDF15*. All confirmed genes were downregulated in FTC and the direction of change agreed between qRT-PCR data and microarray/meta-analysis data. Boxplots of qRT-PCR results for significantly differentially expressed genes are shown on Figure 3. Based on our results, the following genes that most significantly differentiated between FTC/FTA were selected by a meta-analysis: *CPQ (PGCP)*, *PLVAP*, and *TFF3*.

A multivariate ANOVA with two factors: malignancy and oncocytic feature was also performed, in order to evaluate the differential expression between FTC and FTA after adjusting for the effect of oncocytic feature. All seven genes significant in the Student’s *t*-test were also significant in this ANOVA analysis (Table 3). Adding the additional variables such as age, gender, and *RAS* mutation status did not substantially modify the ANOVA results.

### 2.5. Classifier Performance

To evaluate the usefulness of selected genes as diagnostic support, we performed sample classification based on the FFPE dataset. Log-transformation of the gene expression values and a leave-one-out cross-validation of the classifier was performed. In each iteration, the samples were divided into two independent sets: all but one sample were used for significance threshold tuning, gene selection, and classifier training, and the remaining sample was used for testing. Diagonal linear discrimination analysis (DLDA) algorithm was used for the classifier training. After performing all iterations, the classifier’s performance was calculated. The accuracy, sensitivity, and specificity were 78% (95% confidence interval (CI): 67–87%), 76% (95% CI: 56–90%), and 80% (95% CI: 64–91%), respectively. The classifier involved 4 genes with *p*-value below 0.0005 in the Student’s *t*-test, namely *CPQ*, *PLVAP*, *TFF3*, and *ACVRL1*. When accuracy was calculated for non-oncocytic (45 tumors) and oncocytic (24 tumors) tumors separately it was 84% (95% CI: 71–94%) and 67% (95% CI: 45–84%), respectively.

**Figure 4:**
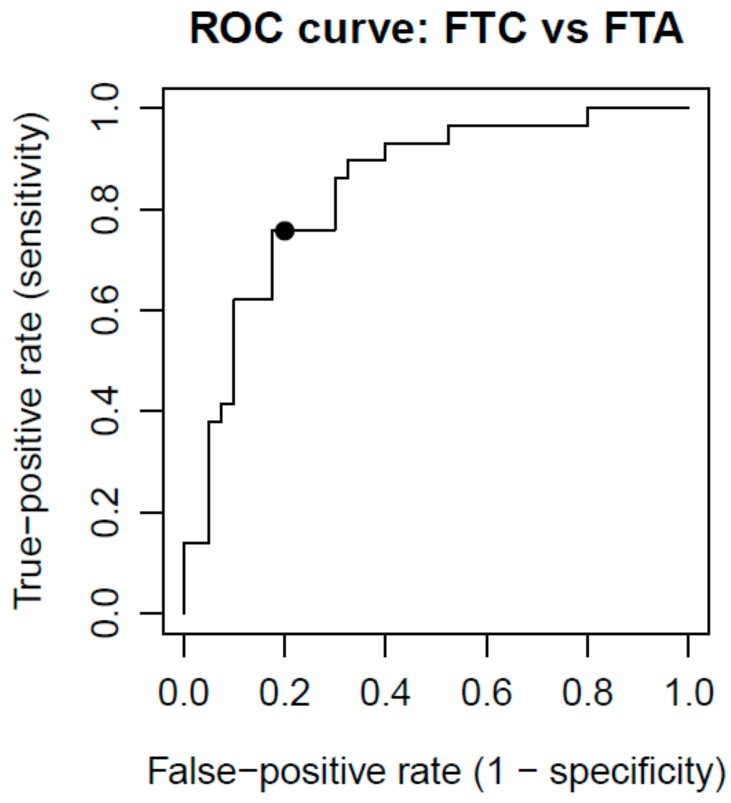
Receiver operating characteristc (ROC) curve analysis for the predictive power of 4-gene classifier, estimated in qRT-PCR dataset. Using a cutoff probability of 50% (marked with black dot), we obtained sensitivity of 76% and specificity of 80%. The calculated area under the ROC curve was 0.84.

A receiver operating characteristic (ROC) curve was also created in order to assess the diagnostic efficacy of the classifier (Figure 4). The area under the ROC curve (AUC) equals 0.84.

### 2.6. RAS Mutation Status

The presence of the *RAS* gene mutation was investigated in freshly frozen FTC (27) and FTA (25) samples. We identified 3 FTC samples with *NRAS* codon 61 mutation and 1 with *KRAS* codon 61 mutation (in total 14.8%). In the FTA samples, we identified only 1 mutated sample with *NRAS* codon 61 mutation (4%) (Table S2). The frequencies of *RAS* gene mutations in malignant and benign samples did not differ significantly.

The status of the *RAS* gene mutations was also analysed in FFPE specimens, however due to limitations related to sample quantity, 14 samples were not fully profiled (only *NRAS* codon 61 was analysed and mutations were excluded in these samples). Among 31 FFPE FTC specimens, 2 samples with *NRAS* codon 61 mutation and 1 with *HRAS* codon 12 mutation were identified (9.7%) (Table S3). More *RAS* mutations were observed in FFPE FTA samples; however, the difference was not significant. Among 40 FFPE FTA specimens, 3 samples with *NRAS* codon 61 mutations, 1 sample with *KRAS* codon 12 mutation, and 1 with *HRAS* codon 61 mutation were detected (12.5%) (Table S3). However, analysis of the total prevalence of *RAS* mutations in FTC and FTA, regardless of the method used for tissue preservation (FF vs. FFPE) demonstrated that there was no difference in the occurrence of *RAS* mutations between FTC and FTA: 12% and 9.3%, respectively.

## 3. Discussion

The differential diagnostics between FTC and FTA is still challenging, particularly because in a molecular sense these lesions lie on a continuum, with similar molecular profiles. Perhaps the 2nd or 3rd molecular hit converts adenoma to carcinoma [28,29] In our study, we performed a meta-analysis of markers differentiating FTC and FTA to summarise the results obtained over a 15-year period (2000–2014), and described in multiple papers.

We obtained a list of 50 genes that were significantly differentially expressed in concordant direction in two or more such papers. We selected 10 genes from the meta-analysis and positively validated 4 of them: *CPQ*, *PLVAP*, *TFF3*, and *FAM189A2*. While, of the 8 genes selected from our own gene expression microarray dataset, three genes: *ACVRL1*, *CLEC3B*, and *ZFYVE21*, were positively validated by qRT-PCR (Table 3).

Due to small number of *RAS* mutation positive samples, we were not able to establish its influence on the expression of genes selected for qRT-PCR validation (Figures S1 and S2).

Finally, we created a gene classifier involving 4 genes (*CPQ*, *PLVAP*, *TFF3*, and *ACVRL1*) that showed a diagnostic accuracy of 78%, sensitivity of 76%, and specificity of 80% for FTC and FTA differentiation. We are aware that our set of genes requires confirmation by an independent clinical study, similar to the study by Alexander et al. [30], which positively verified the clinical utility of a gene classifier proposed by Chudova et al. [25]. However, there are some important differences between Afirma and our approach. While FNAB-based Afirma classifier, used in a preoperative diagnostics, considered all malignant tumors and differed them from benign ones, our classifier was devoted to discriminate only between FTC and FTA on the basis of postoperative material. We did not consider the results of fine-needle aspiration biopsy (FNAB) at any time during our analyses as well as did not link our results to Bethesda Categories. We hope that our classifier may help in such cases where there is a dilemma in a post-operative diagnostics in FTA/FTC distinction. Thus, our work may not be considered as a kind of confirmation of Afirma results.

Transcription profiling, as a method for selection of gene expression markers for distinguishing follicular neoplasms, has been used for over a decade. However, to date, no powerful molecular markers have been established. Similarly, our previous study did not fully accomplish this goal [27]. Therefore, we decided to strengthen our results by performing a meta-analysis of all available studies related to FTC and FTA differentiation [4,5,6,7,8,9,10,11,12,13,14,15,16,17].

The analysis of genes differentially expressed in FTC and FTA in our own gene expression microarray dataset revealed 56 genes. Genes with higher fold-changes and lower *p* values (Table S4), as well as those related to other types of cancer or tumour aggressiveness were preferably selected for qRT-PCR validation. One of these genes, *ACVRL1* correlated with tumour progression in patients with head and neck cancers [31]; whereas two other genes: *ZFYVE21*, and *CLEC3B* were related to cancer invasiveness [32,33]. Four genes, obtained from the meta-analysis were subsequently positively validated *CPQ*, *PLVAP*, *TFF3*, and *FAM189A2*.

Based on the meta-analysis, it appears that building an accurate classifier to differentiate FTCs from FTAs is impossible, even using a large dataset of follicular tumour samples (365 samples in meta-analysis). Therefore, we propose that an accuracy of approximately 80% constitutes a plausible limit of FTC vs. FTA gene classifier performance when analysis is performed in postoperative formalin-fixed material [27].

Possible reason for not satisfying classifier accuracy is that follicular tumours are too similar at the gene expression level. Another hypothetic possible reason is that FTC and FTA classes may have been incorrectly assigned prior to the microarray experiments. Histopathological diagnosis in case of follicular tumours can be influenced by intraobserver variability [34]. To circumvent this, we involved two experienced pathologists in the diagnostic process. It is possible however, that some minimally invasive FTCs did not yet demonstrate any signs of vascular or capsular invasion, and were classified as FTAs.

We assume that FTCs and FTAs are biologically different as they have different clinical outcomes. We are however aware that to date, histopathology constitutes the best option in differential diagnostics of follicular tumours, but a gene-classifier may provide more information in difficult cases. Therefore, we may try to use classifiers ([27], current classifier) to distinguish FTCs and FTAs without histopathological data (unsupervised approach). The results from an unsupervised approach can then be compared to histopathological evaluation, with focus on cases showing discrepancy between the histopathology and classifier data.

It is possible, that we may not reach better classifier performance because of over-simplification that we applied in our analysis. We assumed that both FTC and FTA tumours are internally homogenous, but quite often they are not and they may encompass different zones of differentiation or different histopathological features [35]. Neither FTA nor FTC are completely similar. Considering diversity of biology we cannot expect to cover the whole biological variance with four genes only.

In the present study we decided not to include PTC, because it demonstrated its own, characteristic gene expression profile [36] and the differences between PTC and FTC were quite intense [37]. We believe that an inclusion of PTC to malignant samples may lead to inadequate conclusions, whereas without PTC the study is much cleaner.

The low number of *RAS*-positive samples did not allow an evaluation of the impact of the *RAS* gene mutations on the gene expression profile. However, *RAS*-positive samples did not cluster differentially compared to samples not carrying mutations based on the unsupervised PCA analysis, which suggests small biological differences (Figures S3 and S4). Interestingly, the prevalence of *RAS* somatic mutations in our own FF FTC dataset was 12%, while other studies show the prevalence of *RAS* mutation at 60% [38]. This result might be attributable to the population in the studied region of Europe. Unfortunately, we were not able to analyse of the *RAS* gene mutations in 14 samples due to limited amount of material.

We are aware that our findings would be more robust if we use a single technique of tissue preservation but to a much larger group and the using of FFPE material for validation had a possible limitation. Performing gene expression on FFPE is very challenging and these results could even improve when using cryopreserved samples instead. However, malignant follicular thyroid neoplasms are rare and we had to base on the available material. We did our best to collect as large group as it was possible. We used qRT-PCR with multiple reference genes, to assure that we can amplify sequences coming from reference genes in our tumor samples. Moreover, the results obtained in our study were validated on the independent set of samples. We believe that our results constitute an essential input into the better understanding of molecular biology of follicular thyroid neoplasms. 

## 4. Materials and Methods

### 4.1. Material

#### 4.1.1. Clinical Materials for Gene Expression Microarray Analysis Using Our Own Thyroid Samples

Fresh-frozen (FF) material from 52 tumours (27 FTC, 25 FTA) was used for our gene expression microarray experiments. The samples and microarray data have been already used in our previous studies and are reused in the current study [18,27]. Surgical procedures on patients were conducted in Polish and German centres, at the MSC Institute—Oncology Center in Gliwice, University of Leipzig, University of Halle, and Mainz University Hospital. Samples collected in hospitals were subsequently sent to our laboratory in Gliwice for microarray molecular profiling. Because the diagnosis of follicular thyroid tumours may be often equivocal [34], we attempted to obtain the evaluation of each pathology slide by two pathologists. However, we had access to the paraffin slides in only a part of the samples. If the slide was available for us, the sample was evaluated by two highly qualified pathologists. If the slide was not available for us, we based on the primary diagnosis, stated in the origin hospital by a single pathologist.

Next, the clinical material was divided into primary and secondary sets of tumors, depending on the concordance in histopathological diagnosis. The primary set contained all samples that were independently and concordantly diagnosed by two thyroid pathology experts (Dariusz Lange, Gliwice, and Steffen Hauptmann, Halle (Saale)). The secondary set contained samples that were diagnosed by only one expert, equivocal samples diagnosed by two experts and one sample that was discordantly diagnosed according to malignancy. A description of the material and the frequency of oncocytic tumors is shown in Table 4 (detailed description is given in Table S2).

The study was approved by the local ethics committees (Bioethics Committee of MSC Institute—Oncology Center in Gliwice; approvals: DK/ZMN-493-1-10/09, 20 November 2002 and KB/492-17/11, 9 February 2011), and informed consent was obtained from all patients.

#### 4.1.2. Clinical Materials for Validation Studies

FFPE tissue was used for validation in qRT-PCR experiments. The FFPE tissue consisted of 40 FTA and 31 FTC samples from patients treated in the MSC Institute—Oncology Center in Gliwice. The same set of samples was used in our previous study [27]. Diagnosis of FFPE tumours was based on the independent diagnoses of two pathologists. Material description and frequency of oncocytic tumours is presented in Table 5 (detailed description is given in Table S3 in Supplementary Material). Fresh frozen and FFPE datasets were independent datasets; there was no patient overlap between them.

#### 4.1.3. *RAS* Mutation Screening

All 123 samples of thyroid follicular tumour used for gene expression microarray (52 samples) and qRT-PCR experiments (71 samples) were screened for *RAS* mutations using the Sanger sequencing method with the ABI 3130*xl* Genetic Analyzer. Three *RAS* genes (*H*-, *K*-, *N-RAS*) sequences in commonly mutated codon sites (12, 13, and 61) were analysed. Different primer sets (different size of amplicon) for FF and FFPE samples were used due to sample degradation in FFPE samples (details Table S5).

### 4.2. Gene Expression Microarray-Based Analysis of Our Own Follicular Tumours

#### 4.2.1. Gene Expression Microarray Experiment

FF materials from 52 follicular thyroid tumours (27 FTC, 25 FTA) were used for microarray analysis. RNA was isolated using the RNeasy Mini kit (Qiagen, Hilden, Germany). The RNA quality was assessed with capillary electrophoresis (Bioanalyzer 2100) and all the samples had the RNA integrity number (RIN) higher than 7. An Affymetrix (Santa Clara, CA, USA) HG-U133 PLUS 2 array experiment was performed as described previously [27].

#### 4.2.2. Gene Expression Microarray Data Preprocessing

All microarray data analyses were performed in an R/Bioconductor environment. The microarray data preprocessing was performed in the same way as described in our previous study [27]. The quality of the microarray data was analysed using arrayMvout 1.12.0 library [39]. The raw data were preprocessed using the GCRMA method [40]. The microarray data discussed in this publication have been deposited in NCBI’s Gene Expression Omnibus [41], and are accessible through GEO Series accession number GSE82208 (available online: https://www.ncbi.nlm.nih.gov/geo/query/acc.cgi?acc=GSE82208).

#### 4.2.3. Supervised Analysis of Our Own Gene Expression Microarray Data

The selection of differentially expressed genes was performed independently on the primary and secondary microarray dataset (FF material) (Figure 1), in order to take into account the different levels of diagnosis certainty in the two sets. The following criteria were used for the primary dataset: normalized mean expression of the gene above 4.5, the variance of the gene above the 20th percentile, *p*-value in Student’s *t*-test below 0.001, a fold-change above 1.5 in either direction of the change. The following, less strict criterion was used for the secondary dataset: *p*-value in Student’s *t*-test < 0.05.

For genes selected for validation study, an additional analysis was performed in order to assess the significance of difference between FTC and FTA in microarray dataset comprised of non-oncocytic samples only. The genes were considered significant if the unadjusted *p*-value in the Student’s *t*-test was below 0.005 in primary dataset and below 0.05 in secondary dataset.

In order to adjust *p*-values for multiple comparisons, false discovery rate (FDR) was estimated by Benjamini and Hochberg procedure [42].

### 4.3. Meta-Analysis of All Published Papers

The meta-analysis included all 14 papers in which the difference in gene expression between FTC and FTA was assessed by a high throughput method (gene expression microarrays, SAGE, HDSS, ATAC-PCR); which were published during 2000–2014; and found in PubMed, Google Scholar, or by screening the reference lists of selected papers (Table S1). The following criteria were used for the selection of papers: “follicular thyroid carcinoma/cancer/tumour/adenoma AND microarray/gene expression”.

The lists of genes that were reported by the authors as differentially expressed between FTC and FTA, were extracted from each paper. Different types of gene identifiers were used in each study, such as gene symbols, gene names, GenBank accession numbers, cDNA sequences, Affymetrix identifiers, RefSeq accession numbers, and UniGene accession numbers. All gene identifiers were converted to EntrezID, the lists of genes were compared, and common genes were extracted. Finally, ten genes among the most frequently occurring ones were chosen for qRT-PCR validation (Figure 1).

#### Principal Component Analysis of Microarrays Based on the Meta-Analysis Identified Genes

To visually inspect whether the genes selected in the meta-analysis are able to separate FTC and FTA on an independent dataset, Principal Component Analysis (PCA) was conducted. We performed PCA on our own microarray samples, based on genes that occurred at least once in the meta-analysis (Figure 2 upper plot). We also performed PCA on these samples, based on the genes that occurred at least twice in the meta-analysis (Figure 2 lower plot).

### 4.4. qRT-PCR Validation

#### 4.4.1. qRT-PCR Experiment

FFPE materials from 71 follicular thyroid tumours (31 FTC, 40 FTA) were used for qRT-PCR analysis. RNA was isolated using the FFPE RNeasy Mini Kit (Qiagen) from 5 slices of paraffin blocks selected by a histopathologist. qRT-PCR was carried out for 18 genes (gene names given in Table S6, primer probe design given in Table S7). This experiment was performed with the 7900HT Fast Real-Time PCR (Life Technologies, Carlsbad, CA, USA) using Universal Probe Library fluorescent probes (Roche, Basel, Switzerland) and the 5′-nuclease assay, starting from 200 ng of total RNA. All experiments were performed twice. Results were normalised using the Pfaffl method [43] and the GeNorm application [44] with a combination of 3 normalisation genes: *EIF3A* (eukaryotic translation initiation factor 3, subunit A), *EIF5* (eukaryotic translation initiation factor 5), and *HADHA* (hydroxyacyl-CoA dehydrogenase/3-ketoacyl-CoA thiolase/enoyl-CoA hydratase (trifunctional protein), alpha subunit). Obtained normalised relative expression levels were further log-transformed (Figure S5).

Differences between FTC and FTA were tested using the Student’s *t*-test. In addition, two-way analysis of variance (ANOVA) was used in order to adjust for oncocytic feature. False Discovery Rate (FDR) correction was applied and genes with FDR < 0.05 in both analyses were considered as significant.

#### 4.4.2. Classifier Performance

The classifier was created and validated on the FFPE dataset using CMA package [45] in R/Bioconductor environment. The DLDA was used as a classification algorithm. Student’s *t*-test was used for gene selection with significance level threshold tuned over a grid of significance levels. The performance of the classifier was evaluated by the doubly nested leave-one-out cross validation (LOOCV) approach in order to obtain an unbiased estimate of the accuracy [46]. The outer loop was used for estimating the classifier accuracy, and the inner loop was used for optimising the significance level threshold.

The ROC curve was also created to assess the diagnostic efficacy of the classifier (Figure 4). In the outer leave-one-out loop, for each sample, the probability that the sample belongs to the FTC class was calculated, based on DLDA algorithm. Varying the threshold for the probability, the ROC curve was plotted.

## 5. Conclusions

In our study, we have demonstrated that meta-analysis is a valuable method for selecting possible molecular markers. We showed that genes *CPQ*, *PLVAP*, *TFF3*, *ACVRL1*, *ZFYVE21*, *FAM189A2*, and *CLEC3B* are differentially expressed between FTC and FTA. Furthermore, we propose a 4-gene classifier, which discriminates between benign and malignant follicular neoplasms with the accuracy of 78%. Based on our results, we conclude that there might exist a plausible limit of gene classifier accuracy of approximately 80%, when follicular tumors are discriminated based on postoperative formalin-fixed material.

## Figures and Tables

**Table 1:** Differentially expressed genes selected based on analysis of our own microarray dataset.

No.	Gene Symbol	Gene Name	Affy ID	Primary Dataset				Primary Dataset—Evaluation of Non-Oncocytic Samples Only
				FDR Corrected *p*-Value	Mean Expression in FTC	Mean Expression in FTA	Fold-Change	FDR Corrected *p*-Value
1	*ACVRL1*	activin A receptor type II-like 1	226950_at	0.07	5.52	7.02	0.35	0.12
2	*CLEC3B*	C-type lectin domain family 3, member B	205200_at	0.08	7.54	9.52	0.25	0.13
3	*GABARAPL2*	GABA(A) receptor-associated protein-like 2	209046_s_at	0.08	11.05	11.84	0.58	0.15
4	*ZFYVE21*	zinc finger, FYVE domain containing 21	219929_s_at	0.07	7.39	8.67	0.41	0.04
5	*LIMK2*	LIM domain kinase 2	217475_s_at	0.07	4.32	5.84	0.35	0.12
6	*ZMYND11*	zinc finger, MYND domain containing 11	1554159_a_at	0.10	6.60	8.05	0.37	0.15
7	*DIP2B*	DIP2 disco-interacting protein 2 homolog B (Drosophila)	224872_at	0.11	8.23	7.40	1.78	0.16
8	*MAFB*	v-maf musculoaponeurotic fibrosarcoma oncogene homolog B (avian)	222670_s_at	0.08	8.23	9.78	0.34	0.13

The genes were selected for validation from the genes differentially expressed both in primary and secondary microarray set. Values represented in the table are from analysis of the primary microarray data set.

**Table 2:** The results of a meta-analysis of 14 papers, in which differences in gene expression profile between follicular thyroid cancers (FTC) and follicular thyroid adenomas (FTA) were assessed by a high throughput method. Ten genes (highlighted in bold) were selected for our qRT-PCR validation.

No.	Entrez Gene ID	Symbol	Name	Number of Papers	References	Gene Regulation
1	762	*CA4*	carbonic anhydrase IV	3	[5,9,16]	down
**2**	**1959**	***EGR2***	**early growth response 2**	**3**	**[5,14,16]**	**down**
**3**	**9413**	***FAM189A2***	**family with sequence similarity 189, member A2**	**3**	**[5,9,12]**	**down**
4	7881	*KCNAB1*	potassium voltage-gated channel, shaker-related subfamily, beta member 1	3	[6,9,16] Confirmed by us [27]	down
**5**	**10404**	***CPQ***	**carboxypeptidase Q**	**3**	**[9,11,14]**	**down**
**6**	**5172**	***SLC26A4***	**solute carrier family 26 (anion exchanger), member 4**	**3**	**[6,14,16]**	**down**
**7**	**7033**	***TFF3***	**trefoil factor 3 (intestinal)**	**3**	**[5,6,10]**	**down**
8	185	*AGTR1*	angiotensin II receptor, type 1	2	[13,16]	down
9	822	*CAPG*	capping protein (actin filament), gelsolin-like	2	[14,17]	down
10	1306	*COL15A1*	collagen, type XV, alpha 1	2	[5,13]	down
11	1363	*CPE*	carboxypeptidase E	2	[9,17]	down
12	3491	*CYR61*	cysteine-rich, angiogenic inducer, 61	2	[8,16]	down
13	1733	*DIO1*	deiodinase, iodothyronine, type I	2	[6,12]	down
14	11072	*DUSP14*	dual specificity phosphatase 14	2	[5,16]	down
15	129080	*EMID1*	EMI domain containing 1	2	[5,7]	down
16	953	*ENTPD1*	ectonucleoside triphosphate diphosphohydrolase 1	2	[9,14]	down
17	8857	*FCGBP*	Fc fragment of IgG binding protein	2	[5,17]	down
18	2354	*FOSB*	FBJ murine osteosarcoma viral oncogene homolog B	2	[16,17]	down
19	2697	*GJA1*	gap junction protein, alpha 1, 43 kDa	2	[5,11]	down
20	55830	*GLT8D1*	glycosyltransferase 8 domain containing 1	2	[5,11]	down
21	221395	*GPR116*	G protein-coupled receptor 116	2	[5,9]	down
22	3043	*HBB*	hemoglobin, beta	2	[12,15]	down
23	3309	*HSPA5*	heat shock 70 kDa protein 5 (glucose-regulated protein, 78 kDa)	2	[9,17]	down
24	3400	*ID4*	inhibitor of DNA binding 4, dominant negative helix-loop-helix protein	2	[5,8]	down
25	3590	*IL11RA*	interleukin 11 receptor, alpha	2	[5,11]	down
26	9452	*ITM2A*	integral membrane protein 2A	2	[9,16]	down
27	3708	*ITPR1*	inositol 1,4,5-trisphosphate receptor, type 1	2	[5,11]	down
28	3725	*JUN*	jun proto-oncogene	2	[5,16]	down
29	3912	*LAMB1*	laminin, beta 1	2	[5,11]	down
30	744	*MPPED2*	metallophosphoesterase domain containing 2	2	[16,17]	down
31	22795	*NID2*	nidogen 2 (osteonidogen)	2	[5,7]	down
32	3164	*NR4A1*	nuclear receptor subfamily 4, group A, member 1	2	[12,16]	down
33	22925	*PLA2R1*	phospholipase A2 receptor 1, 180 kDa	2	[12,16]	down
**34**	**83483**	***PLVAP***	**plasmalemma vesicle associated protein**	**2**	**[9,13]**	**down**
35	5583	*PRKCH*	protein kinase C, eta	2	[9,14]	down
36	23180	*RFTN1*	raftlin, lipid raft linker 1	2	[5,9]	down
37	8490	*RGS5*	regulator of G-protein signaling 5	2	[9,13]	down
38	6414	*SEPP1*	selenoprotein P, plasma, 1	2	[5,14]	down
39	7038	*TG*	Thyroglobulin	2	[10,17]	down
40	4982	*TNFRSF11B*	tumor necrosis factor receptor superfamily, member 11b	2	[5,11]	down
41	7173	*TPO*	thyroid peroxidase	2	[10,17]	down
**42**	**440**	***ASNS***	**asparagine synthetase (glutamine-hydrolyzing)**	**2**	**[5,9]**	**up**
43	771	*CA12*	carbonic anhydrase XII	2	[5,12]	up
**44**	**1164**	***CKS2***	**CDC28 protein kinase regulatory subunit 2**	**2**	**[16,17]**	**up**
**45**	**1649**	***DDIT3***	**DNA-damage-inducible transcript 3**	**2**	**[5,7]**	**up**
46	2358	*FPR2*	formyl peptide receptor 2	2	[5,11]	up
**47**	**9518**	***GDF15***	**growth differentiation factor 15**	**2**	**[9,17]**	**up**
48	2896	*GRN*	Granulin	2	[4,8]	up
49	3486	*IGFBP3*	insulin-like growth factor binding protein 3	2	[5,10]	up
50	23089	*PEG10*	paternally expressed 10	2	[5,11]	up

Table 2 shows the Entrez ID, gene symbol, gene name, number of papers in which a particular gene occurs, references to the papers, regulation direction (up–up-regulated in FTC; down–down-regulated in FTC).

**Table 3:** Comparison of gene expression between FTC (29 samples) and FTA (40 samples) in qRT-PCR dataset (*t*-test and two-way ANOVA calculated *p*-values corrected for multiple tests by FDR method). FDR corrected *p*-values below 0.05 are highlighted in bold.

No.	Gene	Gene Selection	*t*-Test—FDR Corrected *p*-Value	Fold Change (FTC/FTA)	Two-Way ANOVA—FDR Corrected *p*-Value
1	*ACVRL1*	Microarrays	**0.0017**	0.58	**0.0036**
2	*ZFYVE21*	Microarrays	**0.0024**	0.69	**0.0036**
3	*CLEC3B*	Microarrays	**0.027**	0.75	**0.045**
4	*ZMYND11*	Microarrays	0.068	0.81	0.17
5	*LIMK2*	Microarrays	0.093	0.79	0.17
6	*DIP2B*	Microarrays	0.23	0.86	**0.04**
7	*MAFB*	Microarrays	0.44	0.89	0.56
8	*GABARAPL2*	Microarrays	Amplification not possible in FFPE samples		
9	*CPQ*	Meta-analysis	**0.000001**	0.49	**0.0004**
10	*PLVAP*	Meta-analysis	**0.00001**	0.51	**0.0001**
11	*TFF3*	Meta-analysis	**0.0004**	0.48	**0.0036**
12	*FAM189A2*	Meta-analysis	**0.0094**	0.68	**0.016**
13	*GDF15*	Meta-analysis	0.058	1.49	0.99
14	*CKS2*	Meta-analysis	0.69	1.07	0.94
15	*ASNS*	Meta-analysis	0.90	1.02	0.17
16	*EGR2*	Meta-analysis	0.90	0.97	0.89
17	*DDIT3*	Meta-analysis	Amplification not possible in FFPE samples		
18	*SLC26A4*	Meta-analysis	Amplification not possible in FFPE samples		

Two-way analysis of variance (ANOVA) was used in order to adjust for oncocytic feature and the results are shown in column “two-way ANOVA—FDR corrected *p*-value”.

**Table 4:** Fresh-frozen material used for microarray analysis.

Set	Histotype	Samples	% of Men	Median Age (Years)	Frequency of Oncocytic Tumours	Concordance of Pathologic Diagnosis by 2 Experts
Primary set	FTC	13	38.5%	66	46.2%	100%
	FTA	13	0%	42	15.4%	100%
Secondary set	FTC	14	21.4%	69	7.1%	28.6%
	FTA	12	25%	49.5	0%	75%
Total	-	52	21.2%	60.5	17.3%	75%

**Table 5:** FFPE material used for qRT-PCR validation.

Histopathological Diagnosis	Number of Samples	% of Men	Median Age (Years)	Frequency of Oncocytic Tumours
FTC	31	32.3%	59	61.3%
FTA	40	12.5%	45	15%
Total	71	21.1%	52	35.2%

## Supplementary Materials

Supplementary materials can be found at www.mdpi.com/1422-0067/18/6/1184/s1.

## Abbreviations

ANOVA	Analysis of variance
ATAC-PCR	Adapter-tagged competitive polymerase chain reaction
AUC	Area under the ROC curve
DLDA	Diagonal linear discrimination analysis
FDR	False discovery rate
FF	Fresh-frozen
FFPE	Formaldehyde-fixed paraffin embedded
FNAB	Fine needle aspiration biopsy
FTA	Follicular thyroid adenoma
FTC	Follicular thyroid cancer
HDSS	High-throughput differential screening by serial analysis of gene expression
IQR	Interquartile range
LOOCV	Leave-one-out cross validation
OTC	Oncocytic thyroid carcinoma
PCA	Principal component analysis
Q1	First quartile
Q3	Third quartile
qRT-PCR	Quantitative real-time polymerase chain reaction
RIN	RNA integrity number
ROC	Receiver operating characteristc
SAGE	Serial analysis of gene expression
WHO	World Health Organization
