# Supplementary material for: Gene Expression (mRNA) Markers for Differentiating between Malignant and Benign Follicular Thyroid Tumours
Source: Int J Mol Sci. 2017 Jun 2;18(6):1184. doi: 10.3390/ijms18061184 (PMC5486007; doi:10.3390/ijms18061184)
Supplement: Supplementary file 1 [file ijms-18-01184-s001.pdf]

Supplementary material

Gene Expression (mRNA) Markers for Differentiating between Malignant and Benign Follicular Thyroid Tumours

Bartosz Wojtas [1,2,†], Aleksandra Pfeifer [1,3,†], Malgorzata Oczko-Wojciechowska [1], Jolanta Krajewska [1], Agnieszka Czarniecka [4], Aleksandra Kukulska [1], Markus Eszlinger [5], Thomas Musholt [6], Tomasz Stokowy [1,3,7], Michal Swierniak [1,8], Ewa Stobiecka [9], Ewa Chmielik [9], Dagmara Rusinek [1], Tomasz Tyszkiewicz [1], Monika Halczok [1], Steffen Hauptmann [10], Dariusz Lange [9], Michal Jarzab [11], Ralf Paschke [12] and Barbara Jarzab [1,*]

[1] Department of Nuclear Medicine and Endocrine Oncology, Maria Sklodowska-Curie Institute—Oncology Center, Gliwice Branch, Wybrzeze Armii Krajowej 15, 44-101 Gliwice, Poland;

[2] Laboratory of Molecular Neurobiology, Neurobiology Center, Nencki Institute of Experimental Biology, Pasteura 3, 02-093 Warsaw, Poland

[3] Faculty of Automatic Control, Electronics and Computer Science, Silesian University of Technology, Akademicka 2A, 44-100 Gliwice, Poland

[4] The Oncologic and Reconstructive Surgery Clinic, Maria Sklodowska-Curie Institute—Oncology Center, Gliwice Branch, Wybrzeze Armii Krajowej 15, 44-101 Gliwice, Poland;

[5] Department of Oncology & Arnie Charbonneau Cancer Institute, Cumming School of Medicine, University of Calgary, Calgary, Alberta T2N 4N1

[6] Department of General, Visceral, and Transplantation Surgery, University Medical Center of the Johannes Gutenberg University, D55099 Mainz, Germany

[7] Department of Clinical Science, University of Bergen, 5020 Bergen, Norway

[8] Genomic Medicine, Department of General, Transplant, and Liver Surgery, Medical University of Warsaw, Zwirki i Wigury 61, 02-093 Warsaw, Poland

[9] Tumor Pathology Department, Maria Sklodowska-Curie Institute—Oncology Center, Gliwice Branch, Wybrzeze Armii Krajowej 15, 44-101 Gliwice, Poland

[10] Department of Pathology, Martin Luther University Halle-Wittenberg, 06108 Halle (Saale), Germany

[11] III Department of Radiotherapy and Chemotherapy, Maria Sklodowska-Curie Institute—Oncology Center, Gliwice Branch, Wybrzeze Armii Krajowej 15, 44-101 Gliwice, Poland

[12] Division of Endocrinology, Departments of Medicine, Pathology, Biochemistry & Molecular Biology, and Oncology, and Arnie Charbonneau Cancer Institute, Cumming School of Medicine, University of Calgary, Calgary, Alberta T2N 4N1, Canada

* Correspondence: Barbara.Jarzab@io.gliwice.pl; Tel.: +48-32-278-93-01; Fax: +48-32-231-93-10

† These authors contributed equally to this work.

Material—detailed description

Microarray study

Fresh-frozen (FF) material from 52 tumours (27 FTC, 25 FTA) was used for the microarray study. Surgical procedures on patients were performed in Polish and German centres at the MSC Memorial Cancer Center and Institute of Oncology in Gliwice (28 subjects), University of Leipzig, University of Halle, and Mainz University Hospital (24 subjects). The patient characteristics given below refer to the Gliwice group only. There were 26 women and 2 men in the Gliwice group, 23 diagnosed with FTA and 5 with FTC (among them 3 patients with widely invasive FTC). The mean age at diagnosis was 47.2 years, with a median of 45.5 years (range: 14–72 years). Eight subjects (among them 3 diagnosed with FTC) underwent primary total thyroidectomy, 15 underwent lobectomy, and the remaining 5 underwent thyroid lobectomy with partial or subtotal resection of a contralateral lobe. The mean and median tumour diameter were 27 mm and 23 mm, respectively (range: 10–55 mm). However, the tumour diameter was unknown in 3 patients. Two patients with FTA were also diagnosed with low risk papillary thyroid cancer. Complementary radioiodine (RAI) ablation was carried out in both patients who achieved complete remission. For the FTC patients, distant metastases (bones and lungs) were present at diagnosis in 2 patients, whereas one subject had local recurrence and distant metastases (mediastinum and lungs) 8 years after the initial diagnosis. Two were RAI-refractory, while RAI treatment with a cumulative activity of 500 mCi resulted in FTC stabilisation for another patient. One remaining FTC patient achieved complete remission after surgery and RAI ablation. The mean time to follow-up in the whole group was 3.7 years (range: 0–12 years).

Validation study

Forty patients were diagnosed with follicular thyroid adenoma (FTA subgroup) and 31 with follicular thyroid carcinoma (FTC subgroup).

The FTA subgroup included 35 women and 5 men, with a mean age at diagnosis of 46.3 years and a median of 45 years (range: 19–79 years). Twenty-seven subjects underwent thyroid lobectomy, and 12 underwent total thyroidectomy. No information regarding the extent of surgery was identifiable for one patient. The median and mean tumour diameter were 27.5 mm and 30.2 mm, respectively (range: 10–80 mm). Twelve patients demonstrated no tumour recurrence, and one patient was diagnosed with nodular goitre in a contralateral thyroid lobe. For 27 remaining subjects there were no data related to the further course of the disease. The mean time to follow-up was 2 years (range 0–9 years).

The FTC subgroup included 21 women and 10 men, with a mean age at FTC diagnosis of 57 years, and a median of 59 years (range 24–83 years). Four patients were diagnosed with widely invasive FTC. The mean tumour diameter was 38.3 mm, with a median of 33.5 mm (range 15–110 mm). However, in 13 cases, no information regarding the tumour was provided. T1, T2, T3, and T4 features were diagnosed in 4, 11, 10, and 1 patient, respectively. Five remaining subjects were staged as Tx. Multifocal tumour growth was observed in 11 subjects. Four patients demonstrated lymph node metastases (N1) and 5 distant metastases at FTC onset. Twenty-four patients underwent total

thyroidectomy; among them primary total thyroid resection was carried out in 10 subjects and two stage procedure in 14 subjects. Six patients underwent surgery primarily due to nodular goitre a few years earlier (all of them finally had total thyroidectomy). Palliative surgery was performed in one remaining patient. All but one were treated with RAI. Seventeen subjects received therapeutic RAI activity only once, whereas 7 subjects required 2 courses of RAI ablation. Six patients with disseminated disease were treated with RAI 3 times or more. Seven patients were RAI refractory. Two subjects additionally underwent external beam radiation (one patient as a palliative procedure). One patient was given sorafenib. Finally, complete remission was observed in 18 patients, asymptomatic hyperthyroglobulinaemia in 3 subjects, partial regression in 1 subject, stable disease in 2 patients, and progressive disease in 6 patients. One patient died due to FTC. The mean time to follow-up was 7.4 years (range: 0–13 years).

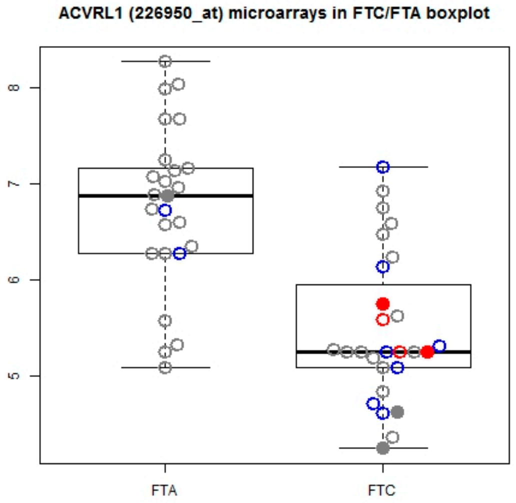
ACVRL1 (226950_at) microarrays in FTC/FTA boxplot
FTA
FTC

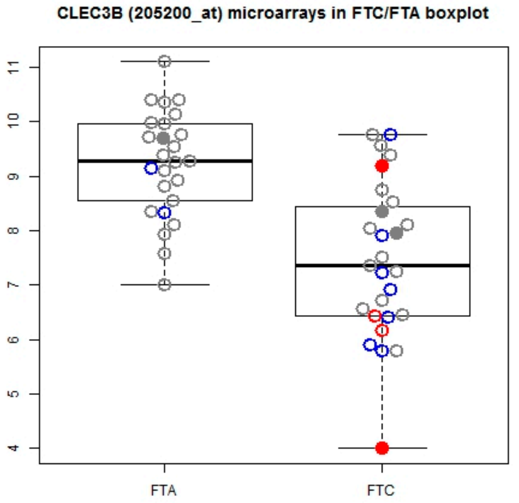
CLEC3B (205200_at) microarrays in FTC/FTA boxplot
FTA
FTC

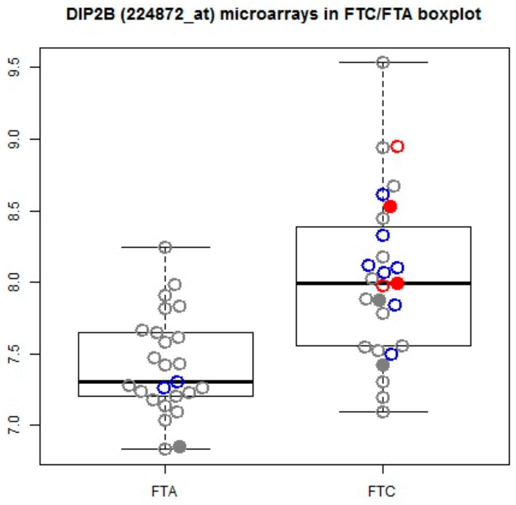
DIP2B (224872_at) microarrays in FTC/FTA boxplot
FTA
FTC

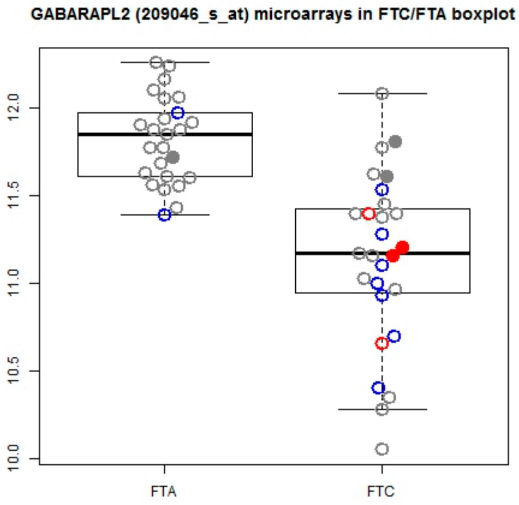
GABARAPL2 (209046_s_at) microarrays in FTC/FTA boxplot
FTA
FTC

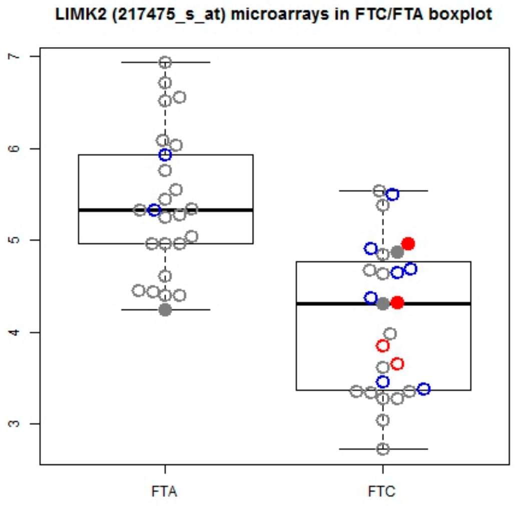
LIMK2 (217475_s_at) microarrays in FTC/FTA boxplot
FTA
FTC

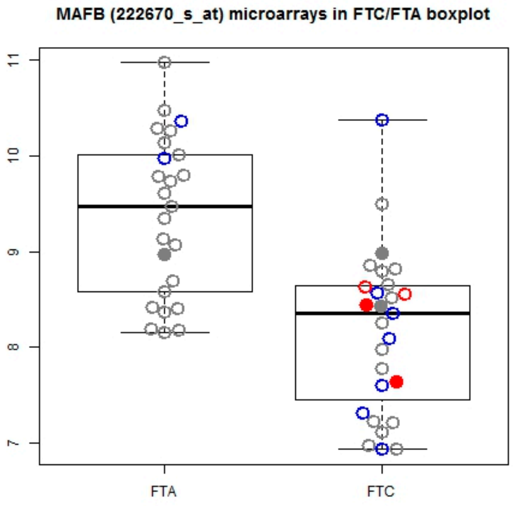
MAFB (222670_s_at) microarrays in FTC/FTA boxplot
FTA
FTC

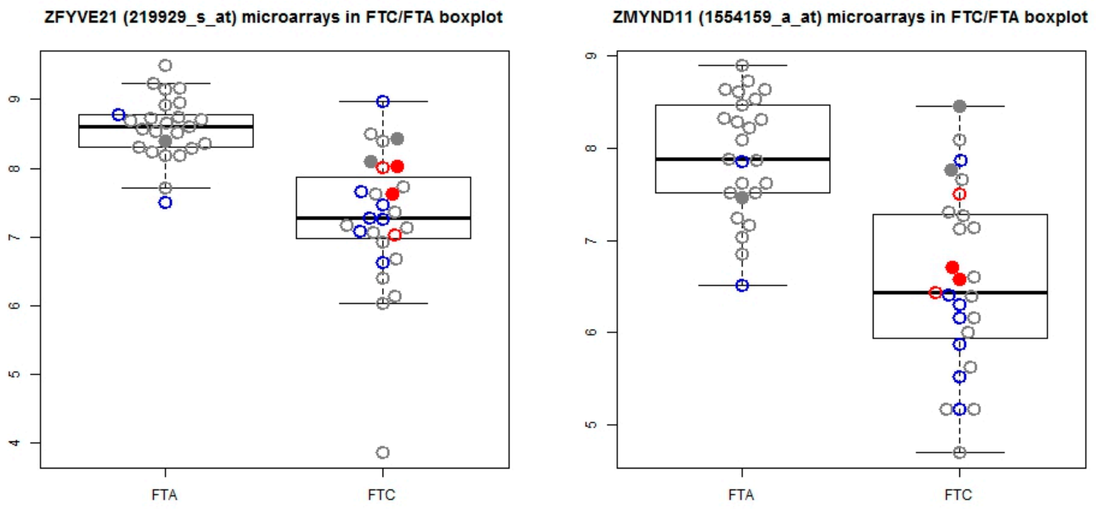

Figure S1. Boxplots from the microarray data set (52 samples, both primary and secondary microarray datasets) with oncocytic and PDTC samples color-coded. Red: PDTC; blue: oncocytic. The solid circles represent samples with RAS mutation.

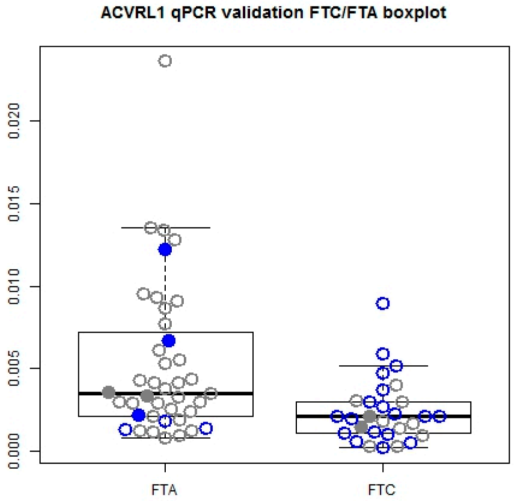
ACVRL1 qPCR validation FTC/FTA boxplot
0.000
0.005
0.010
0.015
0.020
FTA
FTC

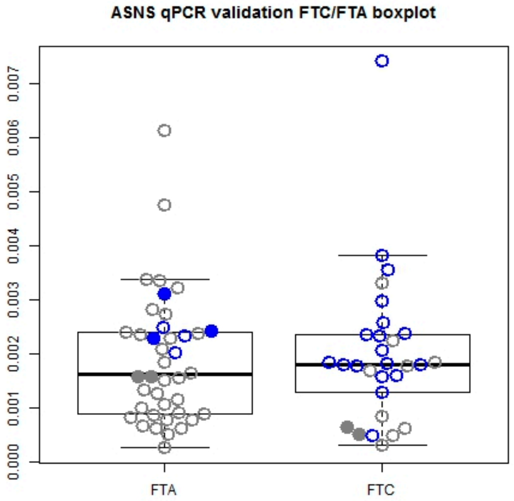
ASNS qPCR validation FTC/FTA boxplot
0.000
0.001
0.002
0.003
0.004
0.005
0.006
0.007
FTA
FTC

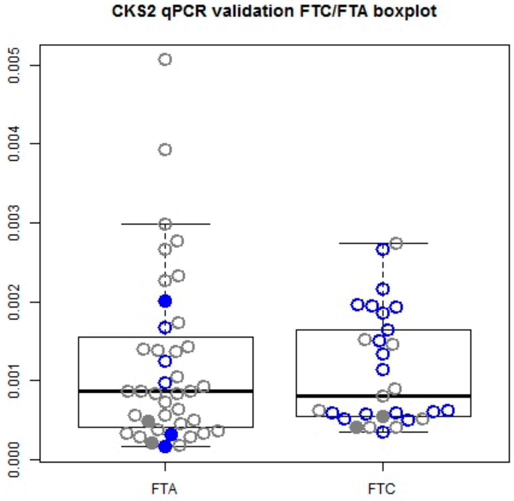
CKS2 qPCR validation FTC/FTA boxplot
0.000
0.001
0.002
0.003
0.004
0.005
FTA
FTC

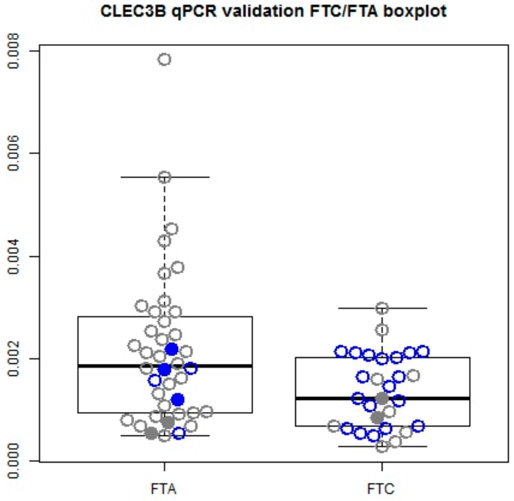
CLEC3B qPCR validation FTC/FTA boxplot
0.000
0.002
0.004
0.006
0.008
FTA
FTC

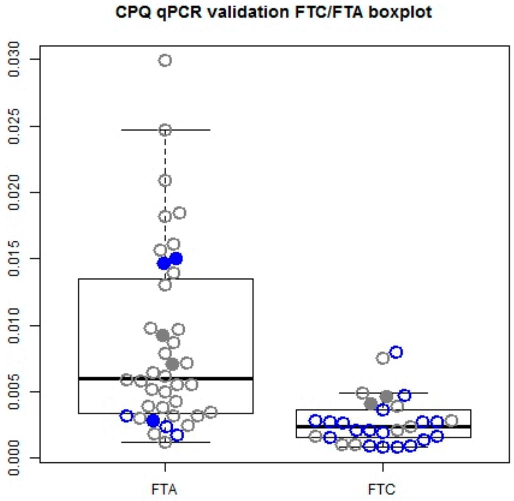
CPQ qPCR validation FTC/FTA boxplot
0.000
0.005
0.010
0.015
0.020
0.025
0.030
FTA
FTC

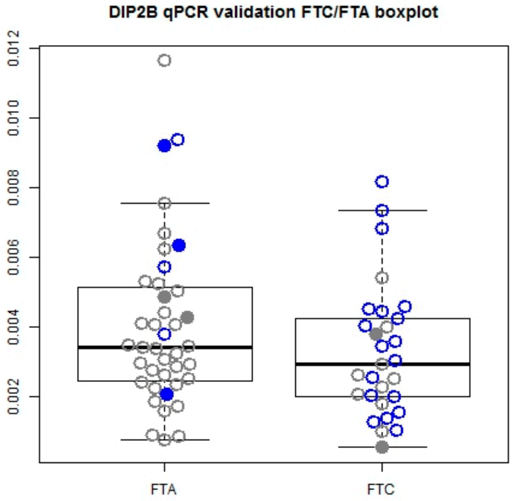
DIP2B qPCR validation FTC/FTA boxplot
0.002
0.004
0.006
0.008
0.010
0.012
FTA
FTC

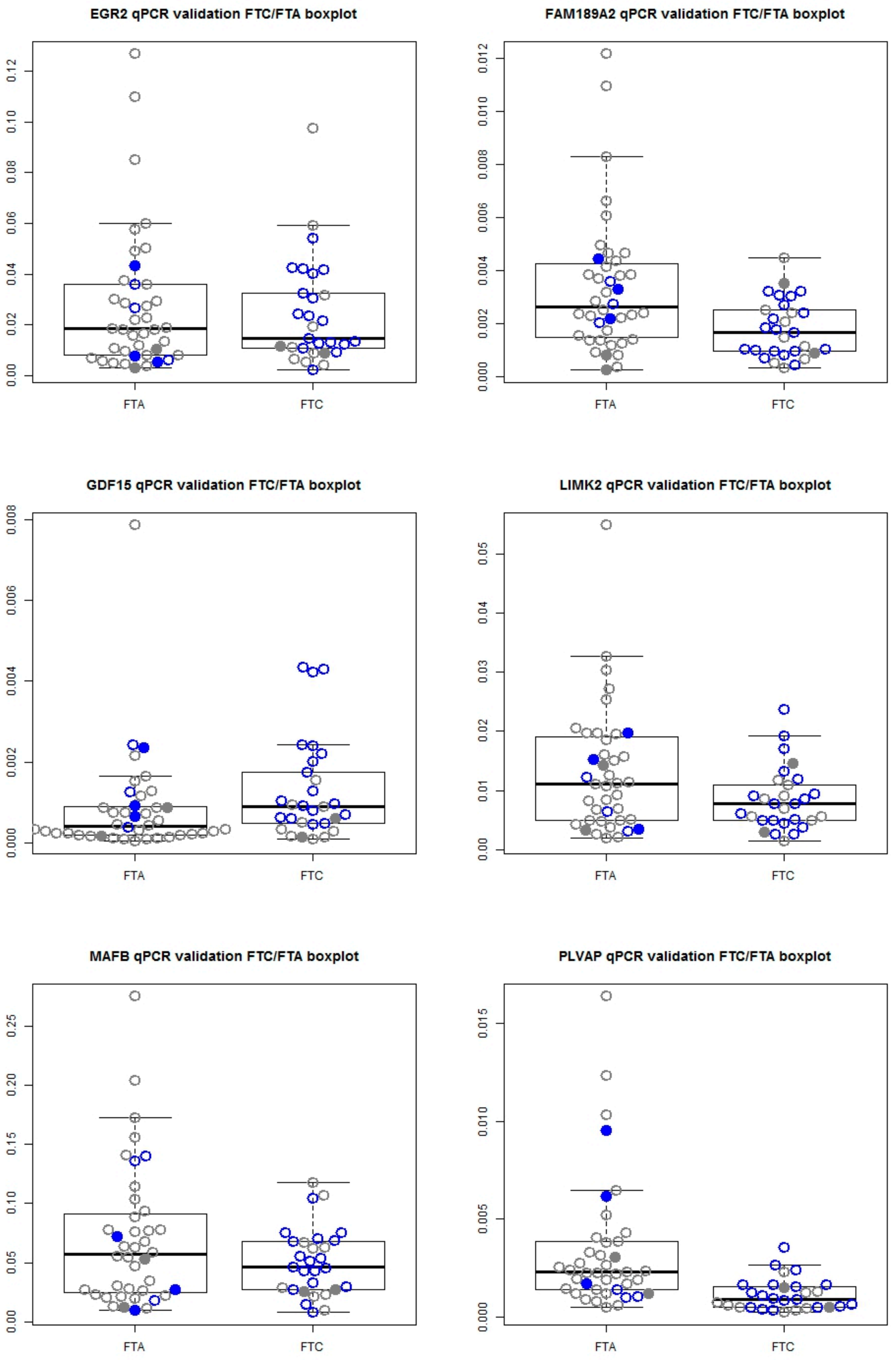
EGR2 qPCR validation FTC/FTA boxplot
0.00
0.02
0.04
0.06
0.08
0.10
0.12
FTA
FTC
FAM189A2 qPCR validation FTC/FTA boxplot
0.000
0.002
0.004
0.006
0.008
0.010
0.012
FTA
FTC
GDF15 qPCR validation FTC/FTA boxplot
0.000
0.002
0.004
0.006
0.008
FTA
FTC
LIMK2 qPCR validation FTC/FTA boxplot
0.00
0.01
0.02
0.03
0.04
0.05
FTA
FTC
MAFB qPCR validation FTC/FTA boxplot
0.00
0.05
0.10
0.15
0.20
0.25
FTA
FTC
PLVAP qPCR validation FTC/FTA boxplot
0.000
0.005
0.010
0.015
FTA
FTC

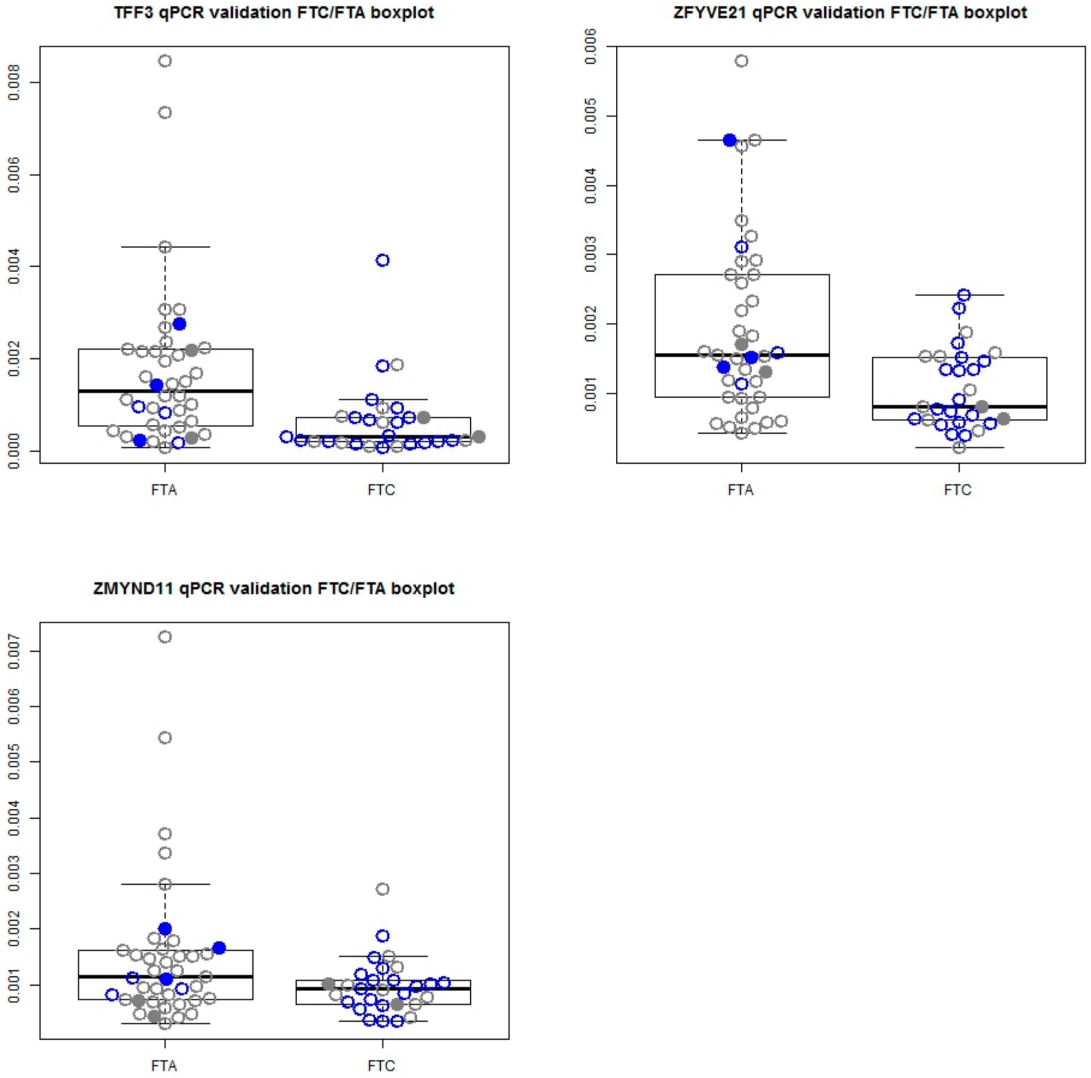

Figure S2. Boxplots from qPCR data (69 samples) with color-coded oncocytic features. Grey: non-oncocytic, blue: oncocytic. The solid circles represent samples with RAS mutation.

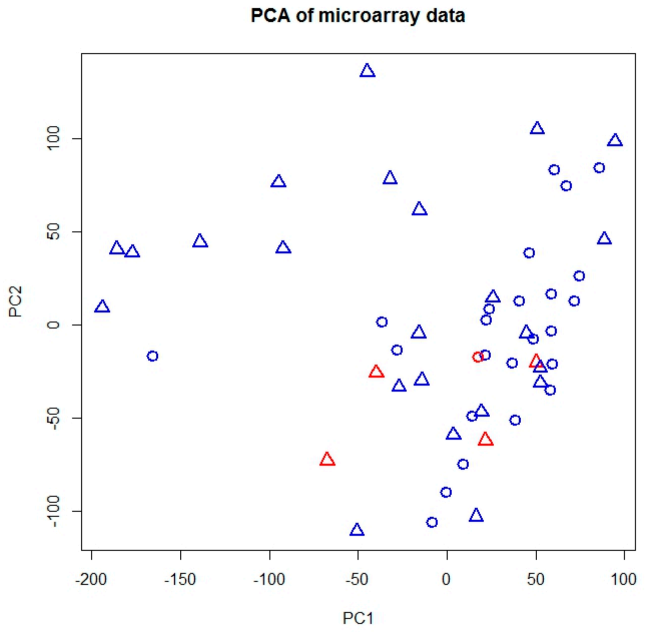

Figure S3. Principal component analysis of microarray data. First and second principal components are plotted. FTC samples are shown as triangles, and FTA samples are shown as circles. Samples with RAS mutation are shown in red; other samples are shown in blue

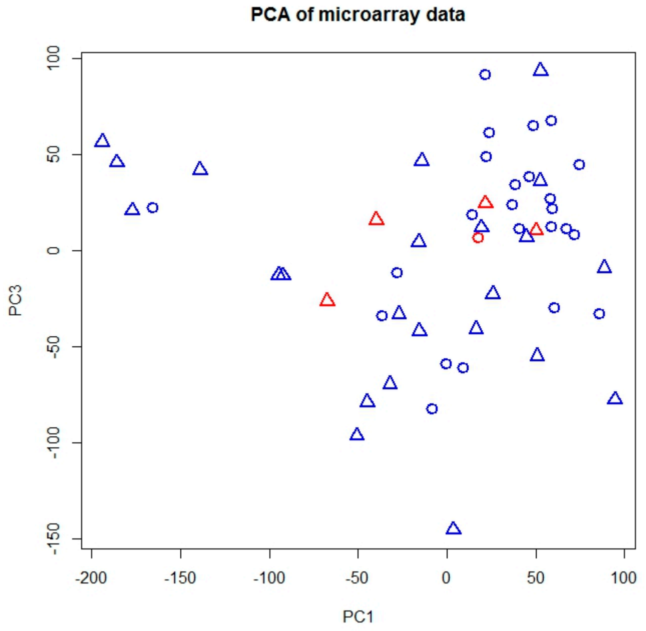

Figure S4. Principal component analysis of microarray data. First and third principal components are plotted. FTC samples are shown as triangles, and FTA samples are shown as circles. Samples with RAS mutation are shown in red; other samples are shown in blue

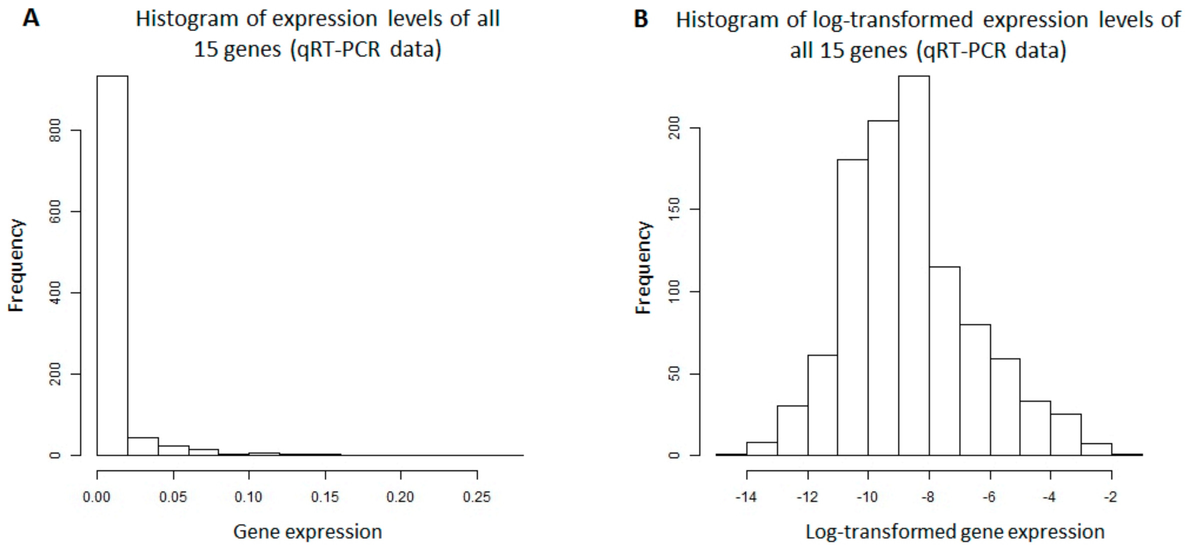

Figure S5. Histograms of qRT-PCR gene expression levels of all 15 genes. A. Normalized relative expression levels. B. Log-transformed normalized relative expression levels.

These histograms enable a comparison of gene expression distribution before and after applying log-transformation.

Table S1. List of papers considered in the meta-analysis

	Reference	Technology	Number of FTA samples	Number of FTC samples	Number of differentiating genes *
1	Takano et al. 2000 [2]	SAGE	1	1	4
2	Barden et al. 2003 [3]	Affymetrix microarray HG-U95	12	7	92
3	Takano et al. 2004 [4]	HDSS	48	29	32
4	Cerutti et al. 2004 [5]	SAGE	1	1	17
5	Chevillard et al. 2004 [6]	cDNA microarrays	4	3	43
6	Weber et al. 2005 [7]	Affymetrix microarray HG-U133A	12	12	77
7	Taniguchi et al. 2005 [8]	ATAC-PCR	45	22	53
8	Lubitz et al. 2005 [9]	Affymetrix microarray HG-U95Av2	14	14	13
9	Fryknas et al. 2006 [10]	cDNA microarrays	10	10	22
10	Stolf et al. 2006 [11]	cDNA microarrays	12	12	30
11	Zhao et al. 2008 [12]	cDNA microarrays	12	12	130
12	Hinsch et al. 2009 [13]	Applied Biosystems Human Genome Survey Microarrays V 2.0	4	8	22
13	Borup et al. 2010 [14]	Affymetrix microarrays HG-U133 Plus 2.0	22	18	100
14	Williams et al. 2011 [15]	Affymetrix HG-U133A	4	15	29
	Total		201	164	

The number of samples describes the samples in the high-throughput gene expression experiments. The number of genes means the number of distinct genes for which it was possible to assign an Entrez ID.

Table S2. Detailed description of freshly frozen samples used for microarray experiment.

SAMPLE	SEX	AGE	ORIGIN	CLASS	SET	onco/PDTC	RAS	DIAGNOSIS
FTC002	F	74	G	FTC	SECONDARY	NO	NO	primary
FTC004	F	80	G	FTC	SECONDARY	NO	NO	primary
FTC005	M	66	G	FTC	SECONDARY	NO	NO	primary
FTC006	F	78	G	FTA	SECONDARY	NO	NO	1 expert
FTC010	F	74	G	FTC	SECONDARY	NO	NO	2 experts
FTC012	M	50	G	FTC	PRIMARY	NO	NO	2 experts
FTC013	F	61	G	FTC	PRIMARY	NO	NO	2 experts
FTC014	F	72	G	FTC	SECONDARY	NO	NO	2 experts
FTC015	F	80	G	FTC	SECONDARY	ONCO	NO	2 experts
FTC016	F	61	G	FTC	SECONDARY	NO	NO	primary
FTC018	M	38	PL	FTA	SECONDARY	NO	NO	primary
FTC020	F	66	PL	FTC	SECONDARY	NO	NO	primary
FTC022	F	47	PL	FTA	SECONDARY	NO	NO	primary
FTC024	F	69	PL	FTC	PRIMARY	NO	K61	2 experts
FTC026	F	44	PL	FTA	SECONDARY	NO	NO	2 experts
FTC028	F	72	PL	FTC	PRIMARY	ONCO	NO	2 experts
FTC030	F	66	PL	FTC	PRIMARY	NO	NO	2 experts
FTC032	F	34	PL	FTA	PRIMARY	NO	NO	2 experts
FTC034	F	39	PL	FTC	SECONDARY	NO	unknown	2 experts (lack of concordance)
FTC036	F	23	PL	FTA	SECONDARY	NO	NO	2 experts
FTC038	F	14	PL	FTA	PRIMARY	NO	NO	2 experts
FTC040	F	42	PL	FTA	PRIMARY	NO	NO	2 experts
FTC042	F	29	PL	FTA	PRIMARY	NO	NO	2 experts
FTC044	F	43	PL	FTA	PRIMARY	NO	NO	2 experts
FTC046	F	31	PL	FTA	PRIMARY	NO	NO	2 experts
FTC048	F	58	PL	FTA	SECONDARY	NO	NO	2 experts
FTC052	F	52	PL	FTA	SECONDARY	NO	NO	2 experts
FTC054	F	60	PL	FTA	PRIMARY	ONCO	NO	2 experts
FTC056	F	69	PL	FTA	PRIMARY	ONCO	NO	2 experts
FTC058	F	60	PL	FTA	SECONDARY	NO	NO	2 experts
FTC060	F	49	PL	FTA	PRIMARY	NO	N61	2 experts
FTC062	M	67	G	FTC	PRIMARY	ONCO	NO	2 experts
FTC066	M	68	G	FTC	PRIMARY	ONCO	NO	2 experts
FTC071	M	72	G	FTC	PRIMARY	PDTC	N61	2 experts
FTC072	F	66	G	FTC	PRIMARY	PDTC	unknown	2 experts
FTC073	M	61	G	FTC	PRIMARY	ONCO	NO	2 experts
FTC074	-	-	G	FTC	SECONDARY	NO	NO	primary
FTC075	-	-	G	FTC	SECONDARY	NO	NO	primary
FTC077	M	76	G	FTC	SECONDARY	PDTC	N61	1 expert
FTC078	M	53	G	FTA	SECONDARY	NO	NO	2 experts
FTC079	F	61	G	FTC	PRIMARY	NO	NO	2 experts
FTC080	M	61	G	FTC	SECONDARY	PDTC	NO	1 expert
FTC081	F	60	G	FTC	PRIMARY	ONCO	NO	2 experts
FTC082	F	71	G	FTC	PRIMARY	ONCO	NO	2 experts
FTC083	F	36	G	FTC	SECONDARY	NO	N61	1 expert
FTC372	F	71	PL	FTA	PRIMARY	NO	NO	2 experts
FTC376	F	55	PL	FTA	PRIMARY	NO	NO	2 experts
FTC381	F	71	PL	FTA	SECONDARY	NO	NO	2 experts
FTC385	M	33	PL	FTA	SECONDARY	NO	NO	2 experts
FTC390	F	33	PL	FTA	PRIMARY	NO	NO	2 experts
FTC392	F	25	PL	FTA	SECONDARY	NO	NO	2 experts

FTC453	F	29	PL	FTA	PRIMARY	NO	NO	2 experts

Table S3. Detailed description of 71 FFPE samples used for qRT-PCR validation experiment.

SAMPLE	SEX	AGE	CLASS	RAS	ONCO
PFA1	F	54	FTA	N61	0
PFA10	F	62	FTA	0	1
PFA104	F	42	FTA	0	0
PFA105	F	23	FTA	0	1
PFA106	F	24	FTA	0	0
PFA11	F	44	FTA	0	1
PFA110	F	74	FTA	0	0
PFA112	M	45	FTA	0	0
PFA113	F	51	FTA	0	0
PFA117	F	74	FTA	0	0
PFA118	F	44	FTA	0	0
PFA120	F	58	FTA	0	0
PFA122	F	25	FTA	unknown	0
PFA13	F	52	FTA	unknown	0
PFA17	F	-	FTA	0	0
PFA19	F	27	FTA	0	0
PFA23	M	57	FTA	0	0
PFA28	F	40	FTA	0	0
PFA3	F	30	FTA	0	0
PFA37	F	42	FTA	unknown	0
PFA38	F	19	FTA	0	0
PFA4	F	79	FTA	K12	1
PFA46	F	68	FTA	unknown	0
PFA54	F	37	FTA	N61	1
PFA6	F	36	FTA	H61	0
PFA62	F	63	FTA	0	0
PFA65	F	21	FTA	0	0
PFA66	F	41	FTA	unknown	0
PFA68	F	52	FTA	unknown	0
PFA73	M	32	FTA	0	0
PFA75	F	48	FTA	0	0
PFA8	F	23	FTA	0	0
PFA80	M	32	FTA	unknown	0
PFA82	F	64	FTA		0
PFA84	F	64	FTA	0	0
PFA85	F	50	FTA	N61	1
PFA9	M	54	FTA	0	0
PFA92	F	43	FTA	0	0
PFA95	F	66	FTA	0	0
PFA96	F	45	FTA	0	0
PFTC13	F	30	FTC	0	0
PFTC14	F	54	FTC	N61	0
PFTC18	F	58	FTC	0	1
PFTC19	M	52	FTC	H12	0
PFTC20	F	54	FTC	0	1
PFTC24	F	59	FTC	0	0
PFTC31	F	65	FTC	0	1
PFTC33	M	44	FTC	0	1
PFTC34	F	53	FTC	0	1
PFTC38	F	49	FTC	0	1
PFTC39 (excluded)	M	75	FTC	N61	0

PFTC4	F	74	FTC	0	0
PFTC40	M	49	FTC	unknown	1
PFTC41 (excluded)	M	78	FTC	0	1
PFTC45	M	62	FTC	0	0
PFTC50	F	62	FTC	0	1
PFTC51	F	66	FTC	unknown	1
PFTC52	F	59	FTC	0	0
PFTC53	F	72	FTC	0	1
PFTC54	M	44	FTC	0	1
PFTC55	M	75	FTC	0	1
PFTC56	F	46	FTC	unknown	0
PFTC58	F	70	FTC	unknown	1
PFTC59	F	41	FTC	0	0
PFTC60	F	83	FTC	unknown	1
PFTC61	F	62	FTC	0	0
PFTC62	F	62	FTC	unknown	1
PFTC63	F	43	FTC	0	1
PFTC65	M	32	FTC	0	1
PFTC8	M	71	FTC	unknown	1
PFTC9	F	24	FTC	0	0

Table S4. Significantly differentially expressed genes based on analysis of our own microarray dataset. Genes marked in grey were selected for QPCR validation.

	**Gene Id**	**Symbol**	**Name**	**p-value in primary data set**	**Fold change in primary data set**	**p-value in secondary data set**	**Fold change in secondary data set**
**1**	**219929_s_at**	**ZFYVE21**	**zinc finger, FYVE domain containing 21**	**8.68E-06**	**0.41**	**1.90E-03**	**0.40**
2	201781_s_at	AIP	aryl hydrocarbon receptor interacting protein	1.29E-05	0.55	7.70E-03	0.63
3	224445_s_at	ZFYVE21	zinc finger, FYVE domain containing 21	2.32E-05	0.50	9.80E-03	0.62
**4**	**217475_s_at**	**LIMK2**	**LIM domain kinase 2**	**2.55E-05**	**0.35**	**3.26E-03**	**0.54**
5	1553313_s_at	SLC5A3	solute carrier family 5 (sodium/myo-inositol cotransporter), member 3	3.32E-05	0.26	8.91E-04	0.35
6	210582_s_at	LIMK2	LIM domain kinase 2	3.59E-05	0.36	8.03E-04	0.58
**7**	**226950_at**	**ACVRL1**	**activin A receptor type II-like 1**	**4.94E-05**	**0.35**	**4.82E-03**	**0.49**
8	200648_s_at	GLUL	glutamate-ammonia ligase	5.05E-05	0.33	3.11E-02	0.48
9	218723_s_at	C13orf15	chromosome 13 open reading frame 15	5.06E-05	0.28	3.27E-03	0.45
10	217543_s_at	MBTPS1	membrane-bound transcription factor peptidase, site 1	5.81E-05	0.49	4.71E-04	0.58
**11**	**209046_s_at**	**GABARAPL2**	**GABA(A) receptor-associated protein-like 2**	**8.30E-05**	**0.58**	**6.35E-04**	**0.68**
12	217202_s_at	GLUL	glutamate-ammonia ligase	9.49E-05	0.34	1.25E-02	0.47
**13**	**222670_s_at**	**MAFB**	**v-maf musculoaponeurotic fibrosarcoma oncogene homolog B (avian)**	**9.56E-05**	**0.34**	**1.03E-02**	**0.56**
**14**	**205200_at**	**CLEC3B**	**C-type lectin domain family 3, member B**	**1.04E-04**	**0.25**	**1.02E-02**	**0.35**

15	203364_s_at	ATG13	ATG13 autophagy related 13 homolog (S. cerevisiae)	1.15E-04	0.52	2.50E-02	0.70
16	214040_s_at	GSN	gelsolin	1.60E-04	0.22	4.09E-03	0.26
17	213164_at	NA	NA	1.64E-04	0.43	6.78E-03	0.49
18	215749_s_at	GORASP1	golgi reassembly stacking protein 1, 65kDa	1.74E-04	0.58	3.56E-02	0.67
19	203240_at	FCGBP	Fc fragment of IgG binding protein	1.77E-04	0.20	3.34E-02	0.30
20	201782_s_at	AIP	aryl hydrocarbon receptor interacting protein	1.90E-04	0.64	2.52E-02	0.80
21	229074_at	EHD4	EH-domain containing 4	1.94E-04	0.36	3.26E-02	0.60
22	211672_s_at	ARPC4	actin related protein 2/3 complex, subunit 4, 20kDa	2.08E-04	0.46	1.61E-02	0.57
23	206113_s_at	RAB5A	RAB5A, member RAS oncogene family	2.22E-04	0.53	3.96E-02	0.61
24	219238_at	PIGV	phosphatidylinositol glycan anchor biosynthesis, class V	2.28E-04	0.50	3.56E-03	0.61
25	209021_x_at	ATG13	ATG13 autophagy related 13 homolog (S. cerevisiae)	2.30E-04	0.63	8.43E-03	0.67
**26**	**1554159_a_at**	**ZMYND11**	**zinc finger, MYND-type containing 11**	**2.65E-04**	**0.37**	**9.49E-04**	**0.42**
27	221619_s_at	MTCH1	mitochondrial carrier 1	2.83E-04	0.50	1.24E-02	0.58
28	204246_s_at	DCTN3	dynactin 3 (p22)	3.17E-04	0.61	1.46E-03	0.62
29	212944_at	SLC5A3	solute carrier family 5 (sodium/myo-inositol cotransporter), member 3	3.20E-04	0.38	3.71E-03	0.43
30	220589_s_at	ITFG2	integrin alpha FG-GAP repeat containing 2	3.21E-04	0.62	9.15E-03	0.56
31	216004_s_at	PKNOX1	PBX/knotted 1 homeobox 1	3.48E-04	0.43	1.17E-02	0.70
32	241874_at	C5orf53	chromosome 5 open reading frame 53	3.79E-04	0.44	1.76E-02	0.54
33	213167_s_at	SLC5A3	solute carrier family 5 (sodium/myo-inositol cotransporter), member 3	3.86E-04	0.20	1.24E-03	0.23
34	215357_s_at	POLDIP3	polymerase (DNA-directed), delta interacting protein 3	4.19E-04	0.41	1.05E-02	0.54
35	224732_at	CHTF8	CTF8, chromosome transmission fidelity factor 8 homolog (S. cerevisiae)	4.24E-04	0.57	2.02E-02	0.72
36	1553243_at	ITIH5	inter-alpha (globulin) inhibitor H5	4.55E-04	0.25	8.88E-03	0.40
37	204049_s_at	PHACTR2	phosphatase and actin regulator 2	4.63E-04	0.46	2.93E-02	0.63
38	211383_s_at	WDR37	WD repeat domain 37	4.65E-04	0.55	1.45E-02	0.78
39	209652_s_at	PGF	placental growth factor	4.68E-04	0.20	2.69E-02	0.35
**40**	**224872_at**	**DIP2B**	**DIP2 disco-interacting protein 2 homolog B (Drosophila)**	**4.89E-04**	**1.78**	**1.98E-02**	**1.33**
41	222507_s_at	TMEM9B	TMEM9 domain family, member B	5.20E-04	0.55	2.36E-03	0.54
42	212890_at	SLC38A10	solute carrier family 38, member 10	5.35E-04	0.53	1.99E-02	0.67
43	233140_s_at	VIPAR	VPS33B interacting protein, apical-basolateral polarity regulator	5.36E-04	0.61	7.34E-03	0.82
44	1554451_s_at	DNAJC14	DnaJ (Hsp40) homolog, subfamily C, member 14	5.57E-04	0.41	2.29E-03	0.49

45	1555434_a_at	SLC39A14	solute carrier family 39 (zinc transporter), member 14	5.69E-04	0.35	8.90E-03	0.36
46	210622_x_at	CDK10	cyclin-dependent kinase 10	5.98E-04	0.27	1.78E-02	0.53
47	231331_at	NA	NA	6.12E-04	0.38	1.55E-02	0.49
48	212914_at	CBX7	chromobox homolog 7	6.38E-04	0.35	1.02E-02	0.48
49	242137_at	RBMS3	RNA binding motif, single stranded interacting protein 3	6.38E-04	0.23	1.95E-02	0.36
50	203188_at	B3GNT1	UDP-GlcNAc:betaGal beta-1,3-N-acetylglucosaminyltransferase 1	6.55E-04	0.50	4.65E-02	0.72
51	213788_s_at	NA	NA	6.93E-04	0.51	3.97E-02	0.70
52	201620_at	MBTPS1	membrane-bound transcription factor peptidase, site 1	6.98E-04	0.51	3.89E-03	0.58
53	222510_s_at	MKRN2	makorin ring finger protein 2	7.13E-04	0.42	1.38E-03	0.53
54	217427_s_at	HIRA	HIR histone cell cycle regulation defective homolog A (S. cerevisiae)	7.19E-04	0.26	5.59E-03	0.44
55	204800_s_at	DHRS12	dehydrogenase/reductase (SDR family) member 12	7.20E-04	0.46	2.62E-04	0.54
56	203222_s_at	TLE1	transducin-like enhancer of split 1 (E(sp1) homolog, Drosophila)	7.79E-04	0.33	1.58E-02	0.46
57	210734_x_at	MAX	MYC associated factor X	7.80E-04	0.54	3.16E-02	0.60
58	202278_s_at	SPTLC1	serine palmitoyltransferase, long chain base subunit 1	7.97E-04	0.45	8.78E-04	0.49
59	220633_s_at	HP1BP3	heterochromatin protein 1, binding protein 3	8.07E-04	0.64	1.11E-02	0.68
60	210524_x_at	NA	NA	8.08E-04	0.28	2.81E-02	0.28
61	200894_s_at	FKBP4	FK506 binding protein 4, 59kDa	8.09E-04	0.50	2.20E-02	0.62
62	204368_at	SLCO2A1	solute carrier organic anion transporter family, member 2A1	8.27E-04	0.37	2.09E-02	0.51
63	233528_s_at	NA	NA	8.89E-04	0.57	1.94E-03	0.55
64	217165_x_at	MT1F	metallothionein 1F	8.94E-04	0.22	9.77E-03	0.22
65	238561_s_at	UTP23	UTP23, small subunit (SSU) processome component, homolog (yeast)	8.98E-04	0.43	1.72E-02	0.60
66	217853_at	TNS3	tensin 3	9.03E-04	0.43	4.84E-03	0.50
67	210144_at	TBC1D22A	TBC1 domain family, member 22A	9.38E-04	0.55	3.03E-02	0.67
68	227615_at	NA	NA	9.49E-04	0.40	4.59E-03	0.49
69	204186_s_at	PPID	peptidylprolyl isomerase D	9.60E-04	0.53	3.32E-03	0.56
70	213629_x_at	MT1F	metallothionein 1F	9.63E-04	0.23	2.22E-02	0.23
71	218559_s_at	MAFB	v-maf musculoaponeurotic fibrosarcoma oncogene homolog B (avian)	9.68E-04	0.37	1.76E-03	0.50
72	203522_at	CCS	copper chaperone for superoxide dismutase	9.76E-04	0.51	9.55E-03	0.63

Table S5. Primer design for RAS mutation profiling. Three first primers marked "_FF" were used for freshly frozen materials and all regions containing 3 mutation sites were amplified. Other primers were used for the FFPE materials.

Gene/primer	Forward primer	Reverse primer	Application	Mutation codons
**HRAS_FF**	5′-CAGGAGACCCTGTAGGAGGA-3′	5′-CTTCACCCGTTTGATCTGCT-3′	cDNA	HRAS 12,13,61
**KRAS_FF**	5′-GGAGAGAGGCCTGCTGAA-3′	5′-AAAGAAAGCCCTCCCCAGT-3′	cDNA	KRAS 12,13,61
**NRAS_FF**	5′-CTGTCCAAAGCAGAGGCAGT-3′	5′-TGGCAAATACACAGAGGAAGC-3′	cDNA	NRAS 12,13,61
HRAS_1213_1	5′-TGAGGAGCGATGACGGAATA-3′	5′-GGGTCGTATTCGTCCACAA-3′	DNA, cDNA	HRAS 12,13
HRAS_1213_2	5′-CAGGAGACCCTGTAGGAGGA-3′	5′-ATCAATGACCACCTGCTTCC-3′	cDNA	HRAS 12,13
KRAS_1213	5′-AGGCCTGCTGAAAATGACTG-3′	5′-TTGGATCATATTCGTCCACAA-3′	DNA	KRAS 12,13
NRAS_1213_1	5′-GCAGTGGAGCTTGAGGTTCT-3′	5′-AAGTGGTTCTGGATTAGCTGGA-3′	cDNA	NRAS 12,13
NRAS_1213_2	5′-TTACTGGTTTCCAACAGGTTCT-3′	5′-CTGGATTGTCAGTGCGCTTT-3′	DNA	NRAS 12,13
HRAS_61	5′-GTGGTCATTGATGGGGAGAC-3′	5′-GCAAACACACACAGGAAGC-3′	DNA, cDNA	HRAS 61
KRAS_61	5′-TGTGTTTCTCCCTTCTCAGGA-3′	5′-TGGCAAATACACAAAGAAAGC-3′	DNA	KRAS 61
NRAS_61	5′-CAAGTGGTTATAGATGGTGAAACC-3′	5′-TCGCCTGTCCTCATGTATTG-3′	DNA, cDNA	NRAS 61

Table S6. Genes selected for qRT-PCR validation

Gene symbol	Gene name
PLVAP	plasmalemma vesicle associated protein
CPQ (PGCP)	carboxypeptidase Q
ACVRL1	activin A receptor type II-like 1
CLEC3B	C-type lectin domain family 3, member B
DIP2B	DIP2 disco-interacting protein 2 homolog B
GABARAPL2	GABA(A) receptor-associated protein-like 2
LIMK2	LIM domain kinase 2
ZFYVE21	zinc finger, FYVE domain containing 21
ZMYND11	zinc finger, MYND-type containing 11
MAFB	v-maf musculoaponeurotic fibrosarcoma oncogene homolog B
EGR2	early growth response 2
FAM189A2	family with sequence similarity 189, member A2
SLC26A4	solute carrier family 26 (anion exchanger), member 4
TFF3	trefoil factor 3 (intestinal)
CKS2	CDC28 protein kinase regulatory subunit 2
GDF15	growth differentiation factor 15
ASNS	asparagine synthetase (glutamine-hydrolyzing)
DDIT3	DNA-damage-inducible transcript 3

Table S7. Primer design for qRT-PCR validation experiment.

**Gene**	**Roche TaqMan Probe**	**Forward primer**	**Reverse primer**
CPQ	#74	5′-gctggagccaagaattcataa-3′	5′-atcgaaagaggtcaccacca-3′
PLVAP	#73	5′-atccctgtagccccatcc-3′	5′-tgtgagcatatccctgcatc-3′
MAFB	#82	5′-agggaagctgccaagctc-3′	5′-atttgaccataagacaaggctgt-3′
ACVRL1	#71	5′-agacccccaccatcccta-3′	5′-cgcatcatctgagctaggc-3′
CLEC3B	#40	5′-ctcaagagccgtctggaca-3′	5′-gtccccttcaggcagacc-3′
DIP2B	#8	5′-catgctgttaaatgccaagg-3′	5′-tcctctctgctcgcttatgaa-3′
GABARAPL2	#70	5′-ccgtcgttgttgttgtgct-3′	5′-ctccacgcatctgtgttcc-3′
LIMK2	#87	5′-caactgcctcatcaagttgg-3′	5′-tccactatgagccgtgacag-3′
ZFYVE21	#22	5′-gggtgtgacccagctgaa-3′	5′-ccgagattcatagaggagcttg-3′
ZMYND11	#42	5′-tgacattgcgaggatgctat-3′	5′-caggacgagcatttgacaag-3′
EGR2	#3	5′-ttataataacactacaccagcaactcc-3′	5′-agacccaactccctcgctac-3′
DDIT3	#9	5′-cagagctggaacctgaggag-3′	5′-tgtttatggctgctttggtg-3′
ASNS	#2	5′-gatgaacttacgcagggttaca-3′	5′-cactctcctcctcggcttt-3′
GDF15	#28	5′-ccggatactcacgccaga-3′	5′-agagatacgcaggtgcaggt-3′
SLC26A4	#84	5′-ggagagcactggaggaaagac-3′	5′-caagaatggcgatcatcaca-3′
FAM189A2	#43	5′-ccctgtgctccatccttct-3′	5′-actgcagccagatccagtct-3′
TFF3	#4	5′-agtgccttggtgtttcaagc-3′	5′-gctggaggtgcctcagaa-3′
CKS2	#25	5′-ttcgacgaacactacgagta-3′	5′-agcctagactctgttggaca-3′
